# A stoichiometric and pseudo kinetic model of loop mediated isothermal amplification

**DOI:** 10.1016/j.csbj.2020.08.020

**Published:** 2020-08-31

**Authors:** Navjot Kaur, Nikhil Thota, Bhushan J. Toley

**Affiliations:** Department of Chemical Engineering, Indian Institute of Science, Bangalore 560012 India

**Keywords:** Loop-mediated isothermal amplification, Stoichiometric model, Molecular diagnostics, Global health, Nucleic acid amplification tests, Reaction network modelling

## Abstract

Loop mediated isothermal amplification (LAMP) is one of the most popular isothermal DNA amplification techniques for research and commercial applications, enabling amplification of both DNA and RNA (with the assistance of reverse transcriptase). The LAMP mechanism is powered by strategic primer design and a strand displacement polymerase, generating products that fold over, creating loops. LAMP leads to generation of products of increasing length over time. These products containing multiple loops are conventionally called cauliflower structures. Existing literature on LAMP provides extremely limited understanding of progression of cascades of reactions involved in the reaction and it is believed that cauliflower structures of increasing length constitute a majority of the product formed in LAMP. This study presents a first of its kind stoichiometric and pseudo kinetic model to comprehend LAMP reactions in deeper depth by (i) classifying LAMP reaction products into uniquely identifiable categories, (ii) generating a condensed reaction network to depict millions of interconnected reactions occurring during LAMP, and (iii) elucidating the pathways for amplicon generation. Despite the inherent limitations of conventional stoichiometric modelling for polymerization type reactions (the network rapidly becomes too large and intractable), our model provides new theoretical understanding of the LAMP reaction pathway. The model shows that while longer length products are formed, it is the smaller length recycle amplicons that contribute more towards the exponential increase in the amount of double stranded DNA. Prediction of concentration of different types of LAMP amplicons will also contribute substantially towards informing design of probe-based assays.

## Highlights

• First of its kind model to comprehend the LAMP reaction network.
• Classification of LAMP reaction products into uniquely identifiable categories.
• Condensed reaction network to depict millions of interconnected reactions.
• Estimation of concentrations of different types of amplicons generated in LAMP.

## Graphical abstract

**Figure:**
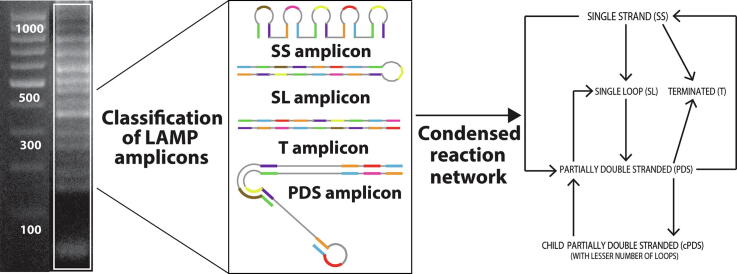

## 1 Introduction

Loop-mediated isothermal amplification (LAMP) has been identified as a powerful isothermal nucleic acid amplification (NAA) technique over the past two decades. It has been a very popular choice among researchers developing isothermal NAA based technologies [1], [2], [3], [4] and has also been recognized as a robust NAA technique for tuberculosis diagnosis by the World Health Organization [5]. A literature search conducted on Web of Science revealed that over the last 5 years, LAMP has been the most frequently used technique for isothermal NAA with ~4x more publications compared to the second most popular technique (ESI Fig. S1). It has recently emerged as a popular technique to enable PCR-free molecular diagnosis of COVID19 [6], [7].#

**Fig. 1:**
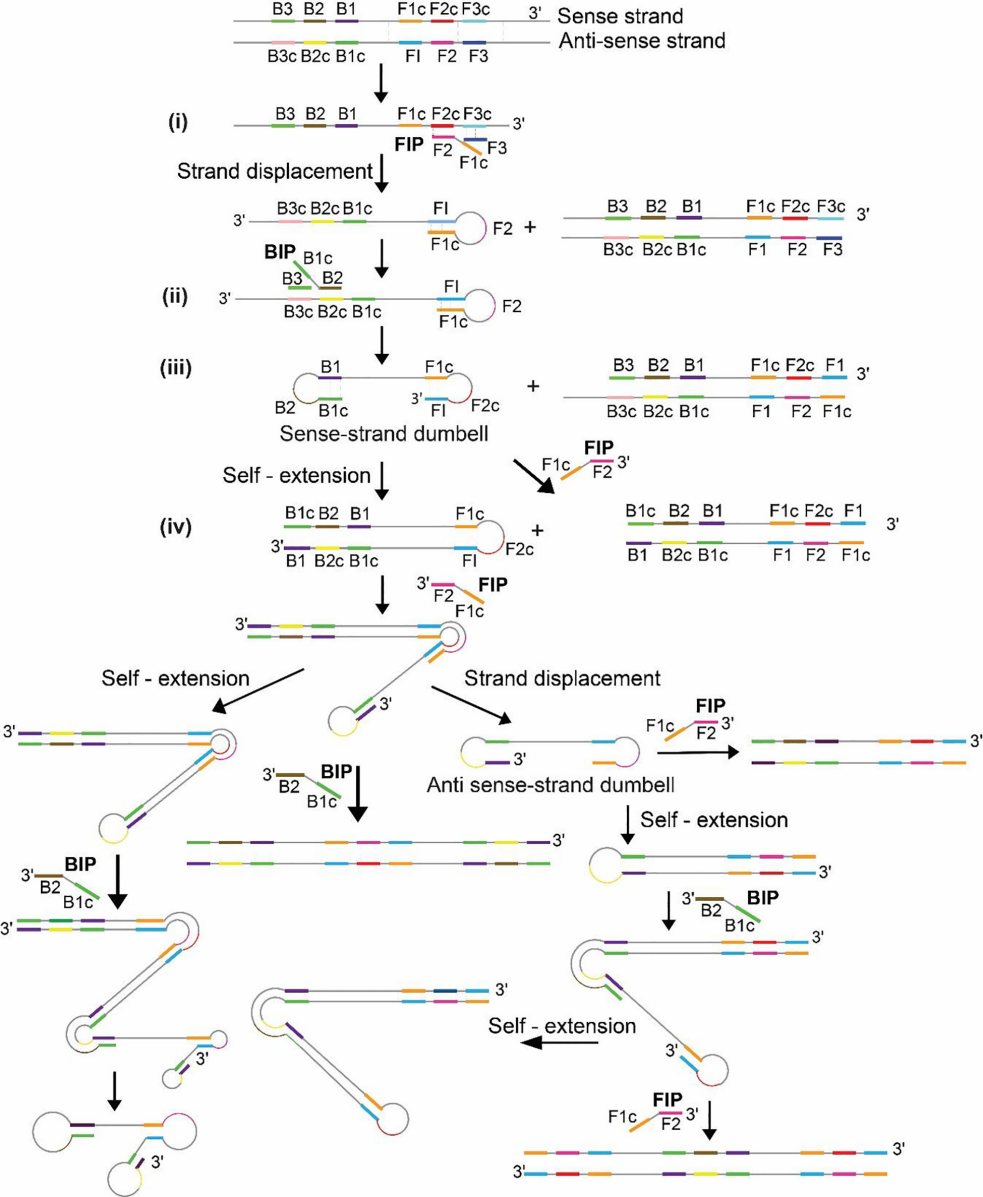
LAMP tree structure. Molecular mechanism of LAMP depicting formation of products of increasing length.

The mechanism for LAMP has been explained in detail by Notomi et al. [13] and some online animations are also available [14], [15]. LAMP, as invented in 2000 by Notomi et al. [13], included four primers – a pair of inner primers (forward inner primer, FIP, and backward inner primer, BIP) and a pair of outer primers (forward outer primer, F3, and backward outer primer, B3). These four primers span six different regions on the target DNA conventionally marked as F1c, F2c, F3c, B1, B2, and B3 on the sense strand of the target DNA as marked in LAMP tree structure (Fig. 1). Briefly, in general the reaction operates at a temperature in the range of 60–65 °C at which the DNA target is spontaneously breathing at multiple locations. This creates regions of weak or broken hydrogen bonds between the two strands of template DNA, creating single-stranded pockets where primers can anneal. FIP partially anneals to its complementary sequence (F2c) on the sense strand and this is followed by extension using a strand displacement polymerase (Fig. 1 (i)). F3 anneals to its complementary sequence (F3c) and extension by the polymerase displaces the newly synthesized strand (Fig. 1 (i)). BIP then anneals to B2c on the newly synthesized strand and is extended by the polymerase (Fig. 1 (ii)). B3 displaces this strand (Fig. 1 (ii)) and a dumbbell structure (Fig. 1 (iii)) is created along with a double-stranded product. The concentration of inner primers in LAMP reactions is usually four to eight times the concentration of outer primers. This ratio of inner and outer primers’ concentration plays a major role in driving LAMP reactions towards formation of the dumbbell structure, which acts as the template required for initiating the exponential phase (Fig. 1 (iv)) of LAMP reactions. In 2002, Nagamine et al. [16] introduced a pair of loop primers in addition to the existing four LAMP primers to accelerate the kinetics and increase the yield of LAMP reactions. Nucleic acid products generated from LAMP are amenable to multiple detection methods [3], [8], [9], [10] and newer fields of research are emerging to enable pathogen detection by coupling LAMP with existing point-of-care diagnostic tools [11], [12].

One of the most noteworthy features of LAMP is the formation of increasing length amplicons, popularly called as cauliflower structures. The mechanism of LAMP, governed by primer sequence design and absence of exonuclease activity in the strand displacement polymerase, results in extension of existing amplicons over time to form products of larger and larger length. This is demonstrated by gel electrophoresis analysis of LAMP amplicons displaying ladder-like patterns containing products ranging from less than 100 base pairs up until the wells in agarose gels. This is in contrast to the gold standard polymerase chain reaction (PCR), in which the amplicon size is known a priori and it is straightforward to estimate the concentration and structure of amplicons formed at the end of the reaction, i.e. when all primers are utilized. Such estimations are not possible for LAMP as LAMP amplicons are not always completely double-stranded and linear. They are rather a combination of intricately coiled single and double-stranded segments, which is confirmed by smudges throughout the gel lanes apart from a few crisp bands at certain specific lengths. This extraordinary element of LAMP reactions creates multiple primer annealing sites on longer length amplicons. The inner primers and loop primers can anneal at numerous locations on each amplicon generating an extremely heterogeneous soup of products. The relatively higher nucleotide incorporation rate of the strand displacement enzyme used in LAMP reactions further aids in maximizing the yield of products. This complex mechanism has limited the theoretical understanding of LAMP reaction pathways.

Most of the available literature on LAMP mechanism depicts a few steps into the exponential stage [17]. There have been theoretical studies to understand the kinetics and mechanisms of PCR [18] and isothermal NAA techniques like isothermal strand displacement amplification (iSDA) [19]. But to the best of our knowledge, detailed studies focussing on mechanistic understanding of generation of amplicons in LAMP do not exist. Extremely limited literature existing on theoretical knowledge about LAMP includes development of a method for better quantification of rise times for LAMP reactions [17], demonstration of the effect of internal primer-template mismatches on LAMP efficiency [20], and observation of the impact of primer dimers and self-amplifying hairpins on LAMP reactions and proposing a thermodynamic parameter to predict non-specific amplification for different sets of LAMP primers [21]. A recent study presented a mathematical model to reduce false-positive diagnosis by LAMP by predicting the expected size of amplicons and comparing it with the results obtained from electropherograms [22]. The authors presented a new method to analyse the increasing length of LAMP amplicons. But they restricted their analysis to only one class of amplicons termed as ‘stem and loop structures’, which as they acknowledge, does not include all different types of amplicons produced during LAMP. Further, their analysis was restricted to products of less than 1500 bp (a maximum of 7 stem and loop structures), whereas LAMP produces amplicons of several thousand base pairs in length. Nonetheless, this study demonstrated the utility of developing a detailed understanding of LAMP mechanism and predicting the sizes of amplicons generated by LAMP.

In this study, we present the first attempt at developing a stoichiometric and pseudo kinetic (SPK) model to provide deeper insights into the cascades of reactions occurring in LAMP with four primers (FIP, BIP, F3 and B3). This was accomplished by first recognizing that the very large number of LAMP amplicons can be categorized into four exclusive structural groups and the complex LAMP reaction mechanism can be condensed into a compact reaction network between these four groups. The different pathways of the condensed reaction network were modelled to develop an SPK model. Given the hundreds of distinct length LAMP products and the stochasticity in their formation, it was not possible to develop a comprehensive kinetic model of LAMP governed by the law of mass action. We therefore developed a “pseudo” kinetic model, i.e. the only time scale in the model emerges from the rate of nucleotide incorporation by the polymerase. The extension rates are considered to be independent of the concentration of free enzyme, nucleotides, and primers. This assumption, in fact, holds true in early stages of the LAMP reaction when there are no limiting reagents. This model presents unprecedented insights into the structures of LAMP amplicons and reaction pathways. It successfully predicts rise times of real-time LAMP amplification curves in close agreement with experimentally measured rise times. The model can be used to predict the concentration profiles of the different amplicon types generated during LAMP, which can inform design of downstream detection strategies.

## 2 Results and discussion

### 2.1 Classification of LAMP amplicons

Analysis of the LAMP tree structure (Fig. 1) with four LAMP primers revealed that there are only two pathways by which a reactant DNA molecule can generate further products: a) primer-based extension, and b) self-extension. Primer-based extension involves the annealing of inner primers (FIP or BIP) to their complementary region on the reactant DNA molecule, followed by extension by DNA polymerase. Self-extension is facilitated by creation of patches of complementary regions within a single DNA molecule. DNA strands loop around to anneal with their complementary sequences and the free 3′ ends are extended by DNA polymerase. Each amplicon formed during LAMP can adopt either of these two strategies for generating subsequent products. The type of reactant and the choice of pathway determines the type of product formed.

**Fig. 2:**
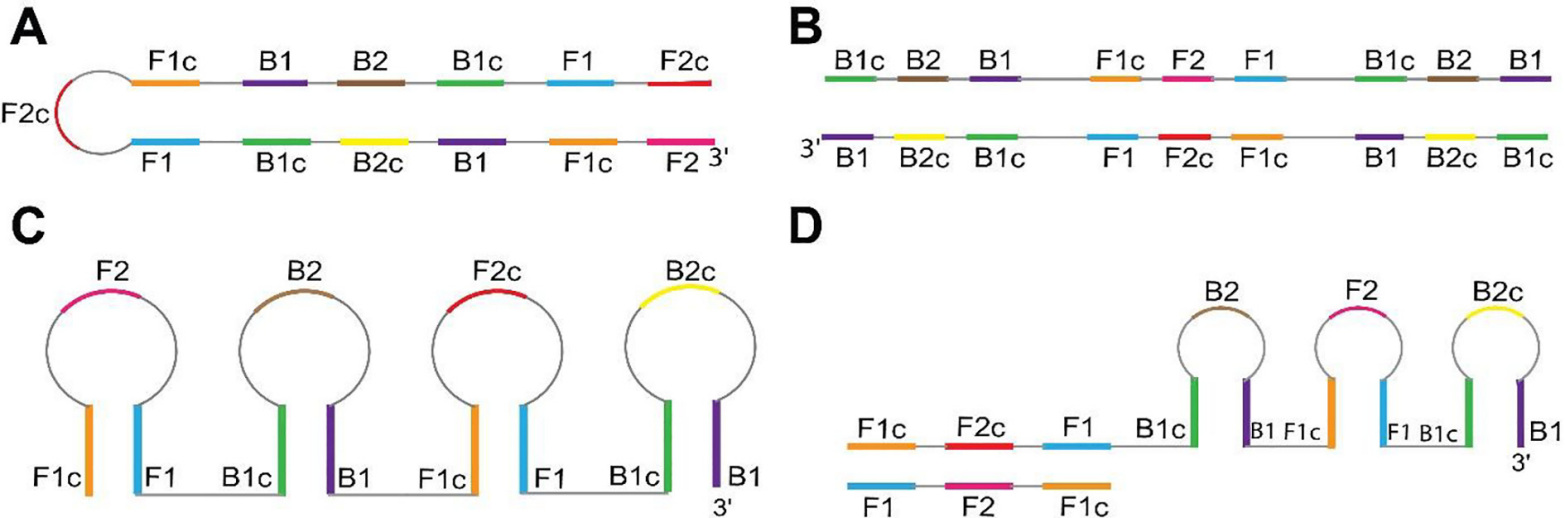
Classification of LAMP amplicons into four categories. (A) Single loop amplicon (SL), (B) Terminated amplicon (T), (C) Single-stranded amplicon (SS), and (D) Partially double-stranded amplicon (PDS).

Careful investigation of literature brought to our attention that a method to classify LAMP amplicons according to their structure does not exist. This motivated us to study all possible structures of LAMP amplicons; we concluded that all LAMP amplicons may be categorized into four structural categories. Fig. 2 shows representative structures for each category of amplicons and the unique identifiers for each category are explained below:

(i) Single-loop (SL) amplicon (Fig. 2A): This amplicon type contains only one single-stranded loop and the remaining part of the amplicon is double-stranded. The single loop of the SL amplicon acts as a primer annealing site.
(ii) Terminated (T) amplicon (Fig. 2B): This amplicon type is entirely double-stranded. We assume that terminated amplicons do not participate in further reactions.
(iii) Single-stranded (SS) amplicon (Fig. 2C): This amplicon type is predominantly single-stranded with short, discontinuous double-stranded sections restricted to B1/B1c and F1/F1c sequences. SS amplicons consist of multiple single-stranded loops that could be sites for potential annealing of primers. The 3′ end of SS amplicons can be extended by the polymerase via the self-extension pathway.
(iv) Partially double-stranded (PDS) amplicons (Fig. 2D): This amplicon type has a combination of single-stranded and double-stranded regions. These amplicons consist of single-stranded regions including multiple loops that could be potential sites for primer annealing along with a continuous stretch of double-stranded section. The 3′ end of PDS amplicons can be extended by the polymerase via the self-extension pathway.

### 2.2 Condensed reaction network

**Fig. 3:**
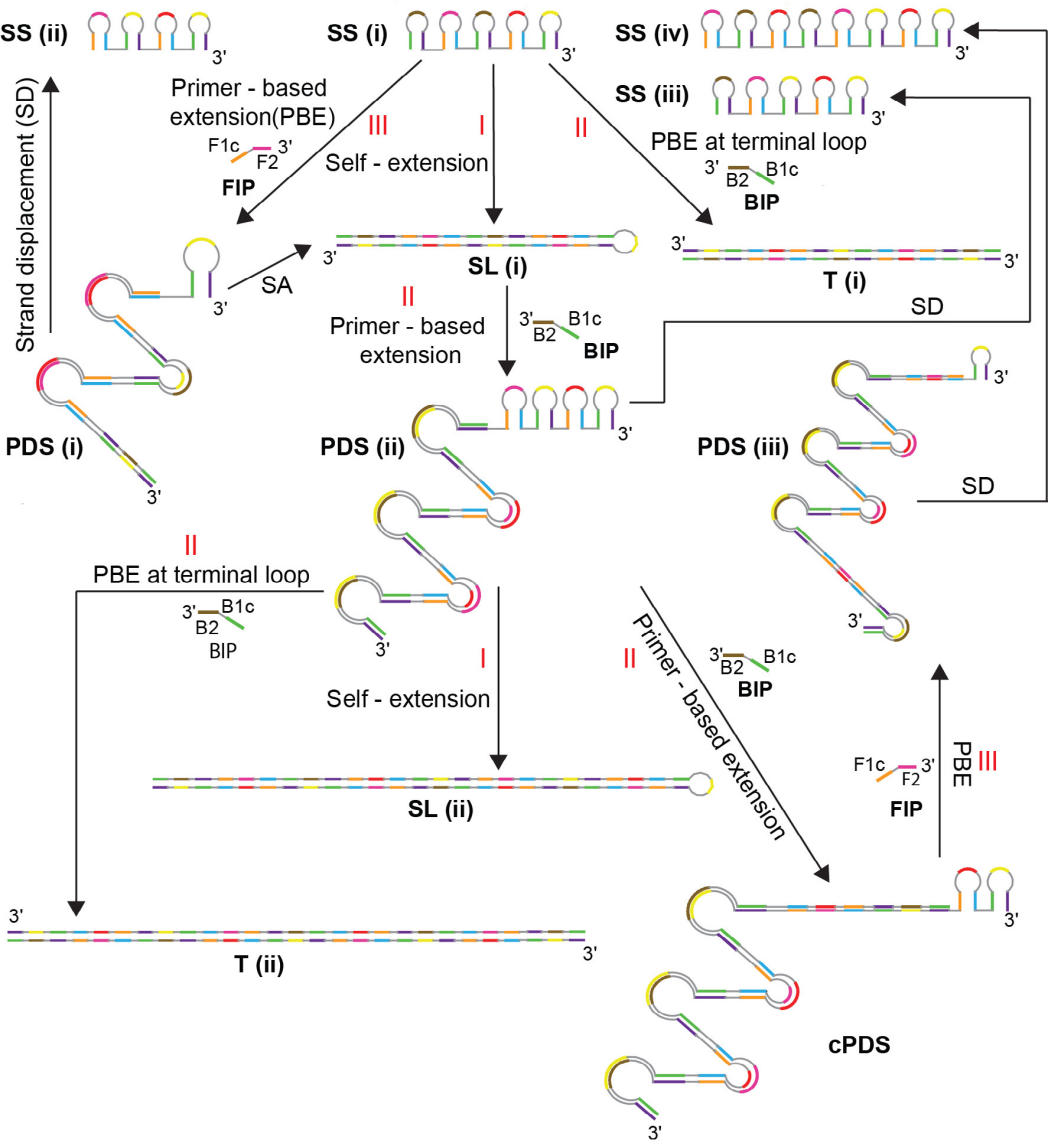
Illustration of all LAMP reaction pathways using amplicon structures. Roman numerals I, II and III represent the different types of reactions each type of amplicon can undergo. PBE refers to primer-based extension.

Another novel feature of this study is creation of a compact representation of the complex LAMP reaction network. We investigated each reaction pathway in detail and found that the expanding reaction network could be expressed as a simplified network of reactions between the four amplicon categories. In order to understand the mechanisms of amplicon generation as captured by the condensed reaction network, consider an illustration for a 5-loop long SS amplicon (Fig. 3 SS(i)) and a LAMP reaction with four primers. The length of SS was chosen to ensure that this reactant SS amplicon and the products formed from it could follow all possible subsequent pathways. For simplification and due to computational challenges, we consider only four primer LAMP reactions for analysis of results and building the model in this manuscript. The classification of amplicons will be the same even on addition of loop primers to the reaction mix (ESI Fig. S2), though the total number of amplicons generated will increase. The fate of each type of amplicon is discussed next.

#### 2.2.1 The SS amplicon: THE SS amplicon has three possible fates:

I) The 3′ end of SS(i) amplicon can undergo self-extension to generate the corresponding SL amplicon (Fig. 3 SL(i)).
II) A primer can anneal at its 3′ end to generate a T amplicon (Fig. 3 T(i)); and
III) A primer can anneal to any loop other than the 3′ terminal loop to generate a PDS amplicon (Fig. 3 PDS (i)). For an SS amplicon to form a PDS amplicon, it should have at least three single loops. An SS with only two single loops will be the dumbbell structure that can only form an SL and a T amplicon.

#### 2.2.2 The SL amplicon:

The SL amplicon (Fig. 3 SL(i)) has a single fate; it can only undergo primer annealing at a single location to generate a PDS amplicon (Fig. 3 PDS(ii)) in which a part of the double-stranded region of the SL opens up to become single-stranded while primer extension creates another section of double-stranded DNA.

#### 2.2.3 The PDS amplicon:

PDS is the most versatile amplicon type as it can form all other types of amplicons. Like SS, it also has three fates:

I) Self-extension of 3′ end of PDS amplicon leads to generation of an SL amplicon (Fig. 3, SL(i) from PDS(i) and SL(ii) from PDS(ii)) along with displacement of an SS amplicon (Fig. 3, SS(ii) from PDS(i), SS(iii) from PDS(ii) and SS(iv) from PDS(iii)). An interesting feature we discovered was that SS(iii) is an exact complement of SS(i) and this helped us create the SS cycle for the SPK model, as will be explained in subsequent sections.
II) Primer annealing to the 3′ end of a PDS leads to formation of T (Fig. 3, T(ii) from PDS(ii)) along with displacement of an SS;
III) Primer annealing at an internal site of a PDS leads to the formation of a sub-class of PDS products that we refer to as child PDS (Fig. 3, cPDS from PDS(ii)) amplicon along with displacement of an SS. For a PDS amplicon to form a cPDS amplicon, it requires at least two single loops and a primer annealing site at any loop except the one at the terminal 3′ end. Primer annealing to the terminal single loop at the 3′ end of a PDS amplicon will generate a T amplicon and not cPDS.

**Fig. 4:**
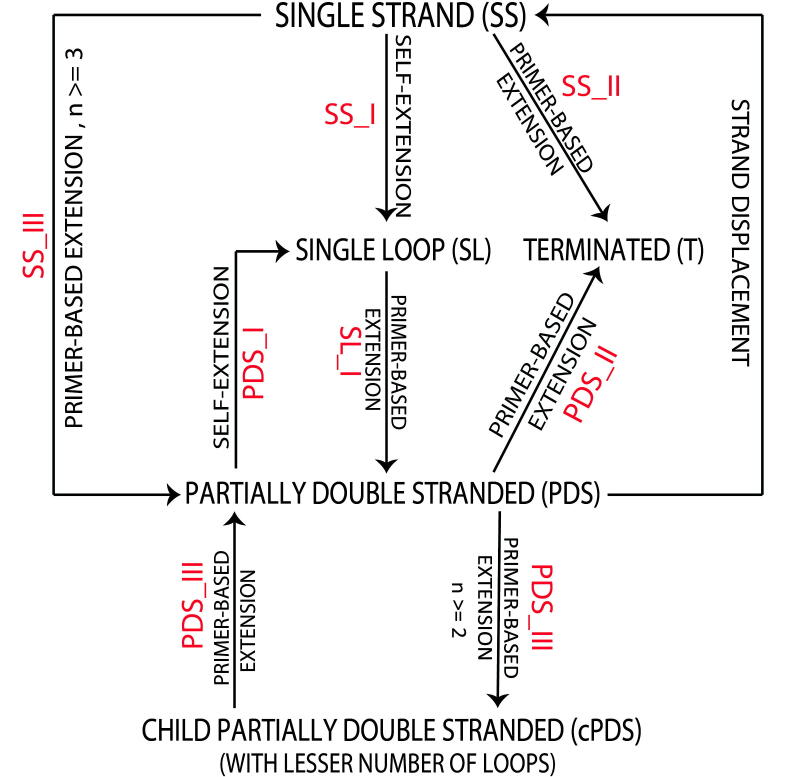
Condensed reaction network for LAMP amplification pathways. Roman numerals I, II and III represent formation of different amplicon types from a particular starting amplicon type.

The abovementioned understanding of the evolution of amplicons with time to generate different categories of products was used to generate a condensed reaction network for LAMP (Fig. 4). SS amplicons can undergo three types of reactions: self-extension (SS_I) to form an SL, primer-based extension (SS_II) to form a T, and primer-based extension (SS_III) to form a PDS. SL amplicons only undergo primer-based extension (SL_I), resulting in formation of PDS amplicons. PDS amplicons can further undergo three types of reactions: self-extension (PDS_I) to form SL amplicons, primer-based extension (PDS_II) to form T, and primer-based extension (PDS_III) to form cPDS amplicons. An SS amplicon is generated by strand displacement whenever a PDS amplicon forms any other product. The suffix I, II and III used in Fig. 4 for different amplicon types are corresponding to the labelling of reaction pathways in Fig. 3 to aid the reader in better understanding of the condensed reaction network.

### 2.3 Brute force stoichiometric and pseudo kinetic model

**Fig. 5:**
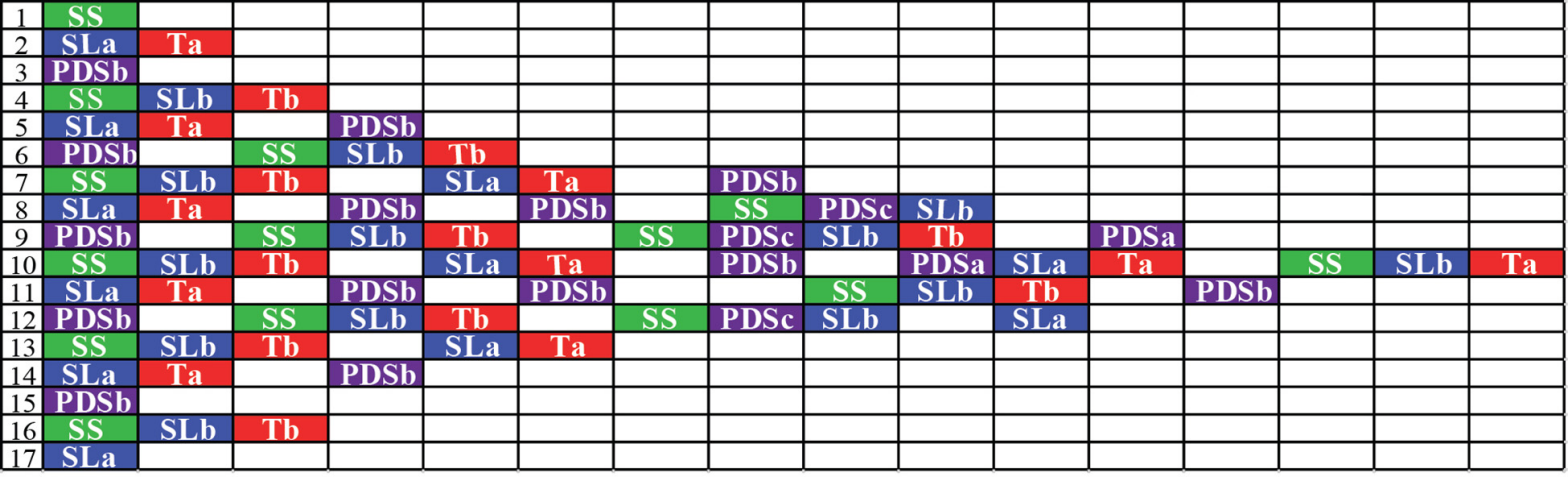
Generation matrix for 100 starting copies of dumbbell and a reaction time of 25 seconds using the brute force model. Amplicon types have been color coded as green for SS, blue for SL, red for T and purple for PDS. Suffix a, b and c help in identifying the type of parent amplicon (SS, SL or PDS, respectively) from which product amplicon is generated. SLa: SL from an SS amplicon, SLb: SL from a PDS amplicon, PDSa: PDS from an SS amplicon, PDSb: PDS from an SL amplicon and PDSc: PDS from another larger PDS amplicon. Ta: T from an SS amplicon, Tb: T from a PDS amplicon. Blank cells separate amplicons generated from different parent amplicons. (For interpretation of the references to color in this figure legend, the reader is referred to the web version of this article.)

The first version of the SPK model was a brute force model that tracked each amplicon molecule individually. It generated all possible products starting from the dumbbell structure, resulting in a growing reaction matrix over time (Fig. 5). Each row of the matrix in Fig. 5 represents one generation. All possible products from reactants in the current row (generation) are shown in the subsequent row. The dumbbell structure is represented by a first-generation SS in the first row (Fig. 5). The dumbbell can only produce two products of type SL and T, which appear in row 2. For ease of tracking, suffixes a, b and c are added to amplicon names depending on whether they are produced from an SS, SL or PDS parent amplicon, respectively. Therefore, the names of amplicons in row 2 are SLa and Ta (generated from an SS). Because Ta does not participate in further reactions and SLa can only generate one product, row 3 only contains a PDSb (generated from SLa). PDSb, in turn, can generate three types of products that show up in row 4 – SS, SLb, and Tb. Note that the SS amplicons are not provided a suffix because they are only formed from PDS amplicons. Now at this stage, there are two amplicons in row 4 that can generate subsequent products. The products formed from these two are separated by a blank cell in row 5. Generation matrix in Fig. 5 (for only 25 s of reaction time) demonstrates the rapid rate at which the number of amplicons having differing sequences and structures increases. Starting with 100 copies of the dumbbell, a total of 77 amplicons are generated in just 25 s. Out of the 77 amplicons, 35 amplicon molecules have unique sequences (6SS, 11SL, 12PDS and 6T). Because this version of the model tracked each amplicon and stored the corresponding data, we faced processing speed and storage issues for reaction time of greater than two minutes. Although the brute force model was computationally too expensive for modelling reactions over meaningful time scales, the data on patterns for amplicon generation obtained from this model helped us understand the reaction pathways better, design methods to circumvent the need of analysing each amplicon individually, and build the current version of the SPK model. Detailed description of the brute force model has been provided in ESI Note S1 and Fig. S3.

### 2.4 Compressed stoichiometric and pseudo kinetic (c-SPK) model for LAMP

**Fig. 6:**
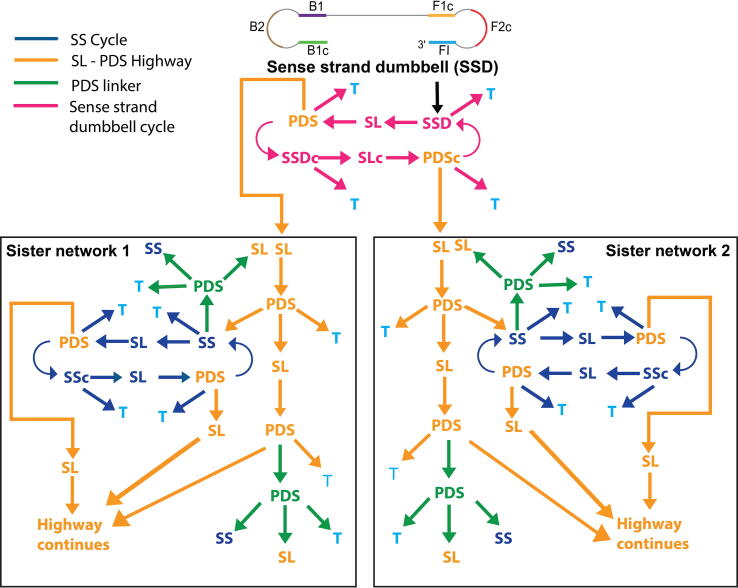
Flow diagram for compressed stoichiometric and pseudo kinetic model. Sense strand dumbbell cycle, SS cycle, SL-PDS highway, and PDS linker capture all the possible reaction pathways for LAMP.

Inferences from the brute force model helped us generate a more refined model which replicated the condensed reaction network better and obviated the need to analyze each amplicon individually. Flow diagram in Fig. 6 explains the crux of the c-SPK model and the assumptions made for constructing this model are explained in ESI Note S2. Starting from the sense-strand dumbbell (SSD) structure, which is a special case of an SS amplicon containing only two loops (and has only two fates as opposed to three; see 2.2.1), we followed all amplicon generation pathways. The first feature of this network is the sense-strand dumbbell cycle (Fig. 6, shown in pink) which handles generation of all SSD amplicons throughout the reaction. We found that SSD is generated at all times during LAMP and each of those SSD molecules act as templates for initiating exponential amplification. In fact, at any stage of the reaction, there are cyclic reaction networks that regenerate products of equal length while simultaneously producing larger products. The larger products participate in additional cycles of equal size product generation while simultaneously generating even larger products, and so on. Once an SSD self-extends it forms an equal length SL amplicon, which has only one fate and extends via primer-based extension to form a longer length PDS (orange colored PDS in SSD cycle). Because this PDS is not the same length as SSD and SL that participated in forming it, this PDS is fed to the SL-PDS highway in sister network 1 (Fig. 6, shown in orange). But when this PDS self-extends to form a longer length SL (orange arrow leading into sister network 1), an SS amplicon (SSDc) is displaced which is complementary and equal in length to SSD. This SSD again generates an equal length SL (SLc) followed by a larger length PDS. The second, larger length PDS is handled by SL-PDS highway in sister network 2. The SS displaced this time is identical to the SSD with which the SSD cycle began and hence this cycle continues over time. Since the first two PDS amplicons formed during SSD cycle form at different points in time, they are handled by two different sister networks to conveniently track the time of formation of all subsequent amplicon types (see ESI Note S3, Note S4 and Fig. S4, S5, S6, S7, S8 and S9 for definition of length of each amplicon type and calculation of number of nucleotides added to form new amplicons in LAMP).

The SL-PDS highway handles increasing length amplicons and whenever a new, larger length PDS is generated, it is fed to the SL-PDS highway. Let us start by following the SL that formed from the PDS in the SSD cycle (SL on the top right corner of Sister Network 1). This SL forms a PDS, which self-extends to form a larger length SL than its parent SL amplicon, displacing an SS amplicon in the process. The SS amplicon is sent to the corresponding SS cycle (Fig. 6, shown in blue) which operates similar to the SSD cycle. Note that the SS formed now has three fates as opposed to the SSD, which only had two. While the SSD cycle exclusively handles SSD amplicons, SS cycles handle all different sized SS amplicons generated during LAMP.

The last pathway is the PDS linker (Fig. 6, shown in green). As explained earlier, SS amplicons can form PDS amplicons and PDS amplicons can form cPDS amplicons. These product PDS amplicons from SS and PDS contain fewer single-stranded loops than their parent amplicon, depending on location of primer annealing. These product PDS amplicons are handled by the PDS linker pathway and can self-extend to form an SL amplicon which is fed to the SL-PDS highway and the displaced SS amplicon creates a new SS cycle. Even though the product PDS amplicons can further form PDS amplicons depending on the number of single-stranded loops in the amplicon, we neglect these scenarios due to a very low contribution of this pathway to the total number of amplicons generated (ESI Note S2, point number 11). T amplicons (Fig. 6, shown in light blue) are formed at different stages of the reaction network; we assume that T amplicons do not participate in any further reactions.

Functions were written to individually model the SS cycle, SL-PDS highway and PDS linker in MATLAB. Data pertaining to an amplicon such as its structure, time of formation (as determined by the rate of nucleotide incorporation), and number of copies were stored in a MATLAB data structure called cell array. A function takes in two cell arrays (titled as data packages, ESI Figs. S10, S11 and S12), one each from sister network 1 and sister network 2. As each function progresses through its respective pathway it performs the process of determining the number of nucleotides added to a reactant to form products (ESI Note S4), time of formation and number of copies (ESI Note S5) of the product amplicon formed from the reactant amplicon. Three small kinetic models (ESI Figs. S13, S14, and S15) were solved to optimize initial concentration of reactants, convert enzyme units to molarity and obtain probability of formation for different product types. The function eventually reaches a stage when the time of formation of the product is greater than a parameter, *t_R_*, the total reaction time. At this point, the abovementioned amplicon details are directed to a matrix which stores details of all the amplicons formed at the end of the reaction time from different functions (SS cycle, SL-PDS highway, PDS linker). A summation of the copies of the latest formed amplicons stored in the matrix provides the total number of copies of products produced for a given reaction time. Similarly, summation of different amplicon types gives the total number of copies of SS, SL and PDS products at the end of reaction time. The program is run for successively increasing values of the parameter, *t_R_* (reaction time) for both the sense strand dumbbell and the anti-sense strand dumbbell for different reaction times. The total copies of amplicons generated for both sense and anti-sense strand is added to generate the total copies versus reaction time curves. This framework has been qualitatively validated in the following section to demonstrate that c-SPK model generates results in agreement with experimentally observed results.

### 2.5 c-SPK model captures characteristic features of LAMP

An important capability of the model is to generate the concentration profile of amplicons and predict rise time (equivalent to threshold cycle (Ct) in PCR) for LAMP reactions. In order to achieve this, the c-SPK model was run for different reaction times starting with 10 and 100 copies of double-stranded DNA to obtain the total copies of amplicons (ESI Note S5) generated for respective reaction times. Because one copy of ds-DNA gives rise to a sense strand and an anti-sense strand dumbbell, and the c-SPK model took starting concentration of the dumbbell as a user-defined input, the starting concentration of the dumbbell was taken as 20 and 200 copies, respectively. Due to limitations in computational power, c-SPK model could be run for limited reaction times (14 min for 20 starting copies and 9 min for 200 starting copies, ESI Fig. S16). In depth description of the computational program and infrastructure used to solve the model is provided in the Methods section and in ESI Note S6. To extrapolate and generate results for greater reaction times, the different reaction time snapshots of concentration profiles were curve fitted using a slight modification of the generalized Richard’s function (Eq. 1). The Richard’s function was successfully used earlier by Subramanian and Gomez [17] for fitting real-time LAMP curves.

(1) $$y\left(t\right)=\frac{k}{1+\exp\left(\left(-b\right)*\left(t-m\right)\right)}$$

where,

*y(t)* represents the concentration of amplicons at time ‘*t*’
*k* represents concentration of amplicons at infinite time
*b* represents the maximum slope of the amplification curve, which occurs at *t* = *m, and*
*m* represents the time at which the growth rate is maximum

Plots for total amplicons versus time, *t*, obtained by solving the c-SPK model for different values of parameter, total reaction time (*t_R_*), were curve-fitted to Richard’s function (ESI Fig. S17), and values of Richard’s function parameters *k*, *b* and *m* were obtained at each value of *t_R_*. The values of *k*, *b* and *m* for 20 (ESI Table S1) and 200 (ESI Table S2) starting copies of the template were then analyzed to obtain time-dependent equations for them (ESI Fig. S18). These equations were then used to develop the methodology for prediction of amplicon concentration–time profiles. ESI Note S6 provides detailed description on curve fitting of model results, creation of time-dependent equations for extrapolation of Richard’s parameters (*k*, *b* and *m*), development of a new technique to compensate the assumption of zeroth order kinetics and introduce the plateau phase into the model generated curves, and shows the results for concentration profiles of amplicons obtained from c-SPK model results. Despite a few simplifying assumptions, rise time predictions from c-SPK model were comparable with experimental results. Rise times predicted by c-SPK model were 96.89 and 81.66 min for 20 and 200 starting copies of the dumbbell, respectively, while the corresponding experimental rise times were 103.76 ± 7.79 (N = 3) minutes and 81.45 ± 2.21 (N = 3) minutes, respectively (ESI Fig. S19).

**Fig. 7:**
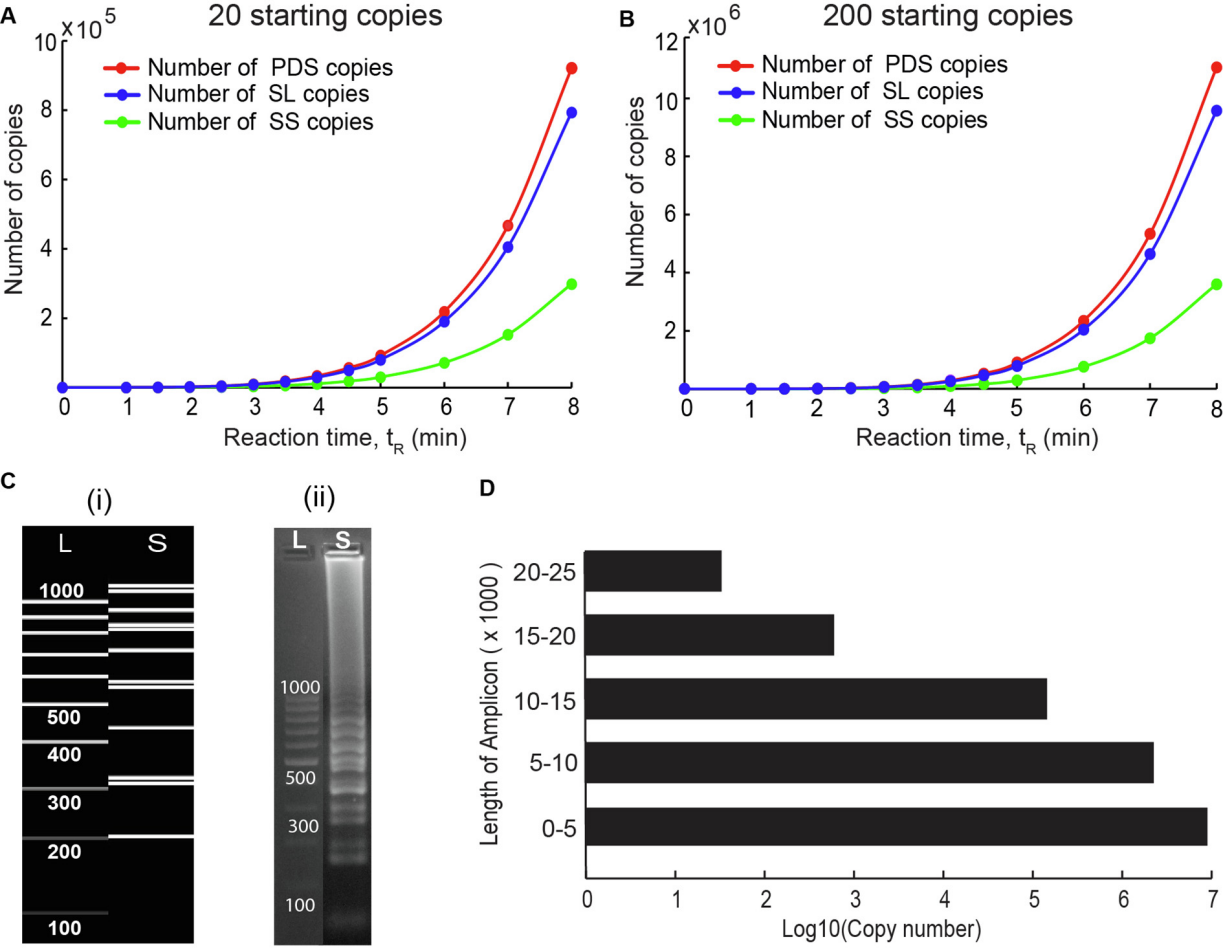
Results from the c-SPK model. (A) Plots for total copies of PDS, SL and SS amplicons versus time for (A) 20 and (B) 200 starting copies of dumbbell for a reaction time of eight minutes. (C) (i) Simulated gel depicting ladder like pattern associated with LAMP amplicons obtained from the model for a reaction time of 7 minutes with 200 starting copies of the dumbbell. (ii) Experimental gel electrophoresis image for LAMP amplicons obtained at the end of 120 minutes reaction starting with 10 copies of double-stranded gDNA. (D) Length pyramid depicting the number of copies formed for different ranges of length of LAMP amplicons for a reaction time of 7 minutes with 200 starting copies of dumbbell.

An even more powerful result from the c-SPK model is prediction of number of copies formed for the different categories of LAMP amplicons (Fig. 7A and B). Because the model identifies different classes of amplicons, it provides amplicon counts for each category, which has been impossible to estimate till date. Following the strategies developed in the previous section, the number of amplicons generated under each category were calculated for various reaction times and starting dumbbell concentrations. Results show that at 8 minutes of reaction time, PDS and SL amplicons form a greater percentage of total amplicons (roughly 85%) while SS and T amplicons constitute the remaining minor fraction (roughly 15%). Since PDS and SL amplicons are predominantly double-stranded and T amplicons are completely double-stranded, we believe that majority of products formed at the end of a LAMP reaction are double stranded. This also explains successful digestion of LAMP amplicons using restriction enzymes, a technique which is widely used to confirm target-specific LAMP amplification. SS amplicons act as intermediate amplicons containing multiple primer annealing sites, leading to formation of other kinds of amplicons during the reaction. Therefore, it would not be a good strategy to design probes for SS amplicons. Probes must be designed for sections of PDS or SL amplicons. While these results are for only 8 minutes reaction time, it is safe to assume that these fractions will be maintained throughout the course of the reaction. These results will empower decisions for downstream detection techniques, for instance design of different types of probe-based detection strategies for LAMP amplicons.

c-SPK model also captures the distinctive feature of ladder-like appearance of LAMP amplicons on gels. All amplicons generated within a reaction time of 7 minutes and 200 starting copies of the dumbbell were considered to create a simulated gel (Fig. 7C (i)). Since LAMP amplicons are transient in nature (keep on forming and being utilized in reaction), reaction time of seven minutes was chosen as it was found to be representative of presence of varied length amplicons. The simulated gel captured the ladder-like appearance of LAMP amplicons as seen in the experimental gels (Fig. 7C(ii)) for LAMP amplicons with 4 primers and a reaction time of 120 minutes. Amplicon bands of different sizes were observed, and their positions were similar to positions of bands in experimental gel. Although a note of caution here would be that while LAMP amplicons are assumed as rigid molecules for length calculations in c-SPK model, in reality LAMP amplicons are cauliflower structures that migrate through the gels at a different rate than fully double stranded DNA. This would explain why the location of bands in experimental gels does not exactly match their linear length. Another significant inference obtained from the c-SPK model is the that while longer length amplicons are formed due to the extension of cauliflower structures, it is the shorter length amplicons that form a major proportion of LAMP products. At the end of 7 minutes, starting with 200 copies (Fig. 7D), the number of amplicons was highest for 0–5000 nucleotides long amplicons followed by 5000–10,000 nucleotides long amplicons followed by 10,000–15,000 nucleotides long amplicons. A drastic reduction in the total number of amplicons was observed for amplicons in the category of 15,000–20,000 nucleotides and 20,000–25,000 nucleotides long amplicons. This can be explained by the fact that the shorter length SS amplicons are formed faster, which can keep giving rise to the same sized products via the SS cycle (Fig. 6) and larger length amplicons via the SL-PDS highway (Fig. 6).

## 3 Conclusion

This work provides the first framework to: (i) classify LAMP reaction products into uniquely identifiable categories, (ii) generate a condensed reaction network to depict the complex cascade of reactions taking place in LAMP, and (iii) understand the amplicon generation pathways involved in LAMP in depth. While this model incorporates many simplifying assumptions and is not an absolute replica of real LAMP reactions, our attempt is to open the field for deeper theoretical understanding of the LAMP reaction network. Improved comprehension of LAMP mechanism would aid in designing robust probe-based detection strategies to enhance specificity for detection of target DNA, replacing traditional whole DNA detection methods fraught with false positive results [23]. Future work in our group will focus on developing sensitive lateral flow detection strategies of LAMP amplicons guided by the results of this model. We would also like to invite researchers from fields of polymer science, population balance modelling, and other related areas to extend application of principles from their fields to understand the growth of complex DNA molecules in LAMP.

## 4 Materials and methods

### 4.1 Experimental methods

The LAMP assay used for comparison of experimental and c-SPK model data was designed for the *hspX* gene of *Mycobacterium tuberculosis*. Primers for the LAMP assay were taken from a published study [24], but only the inner primers (FIP and BIP) and the outer primers (F3 and B3) were used for this work. 12.5 µl real-time LAMP reactions were conducted in a Quant Studio 3 (Applied Biosystems) PCR machine with the following composition: 1.6 µM forward inner primer (FIP) and backward inner primer (BIP), 0.2 µM forward outer primer (F3) and backward outer primer (B3), 1X LAMP reaction buffer, 8 mM MgSO_4_, 0.9 M Betaine (B0300-1VL, Sigma-Aldrich), 2.5 µl dNTP mix (10 mM each, N0447S, New England Biolabs (NEB)), 4 units of *Bst 2.0* WarmStart® DNA polymerase (M0538S, NEB), 1 µl of the target DNA, 3X SYBR Safe I (Invitrogen, P/NS33102) and DEPC water to adjust the final volume to 12.5 µl. LAMP products were also analyzed using gel electrophoresis by running them in 2% agarose gels (115 V) stained with ethidium bromide and the gels were imaged in a custom-made gel imager. Richard’s equation was used to curve fit experimental real-time amplification curves for each sample to obtain Richard parameters *m* and *b*. Time to positive, Tp, was calculated based on the method developed by Subramanian and Gomez [17] using the below formula:

$$\mathrm{Tp}=m-\frac{2}{b}$$

Triplicates were run for both 10 and 100 starting copies of the template. Tp values for replicates were taken to calculate the average and standard deviation for Tpfor 10 and 100 starting copies of template, respectively.

### 4.2 Computational methods

#### 4.2.1 Defining characteristic features of LAMP amplicons

Because increasing length amplicons are produced over time, each category of amplicons has many variants depending on the length of the amplicon. Therefore, it became imperative to define a method of identification for these different types of amplicons. The structure and length of an amplicon were used as its unique identifiers. A detailed description of the representation style used to depict a particular category of amplicons and formulae used to calculate the length of amplicons from each category is provided in ESI Note S3. The procedure used to calculate the number of nucleotides added to reactant amplicons to form the corresponding product amplicons is explained in ESI Note S4.

#### 4.2.2 Compressed stoichiometric and pseudo kinetic (c-SPK) model for LAMP

Drawbacks of the brute force model were overcome by developing the c-SPK model. The set of assumptions involved in this model are provided in ESI Note S2. The only time scale in the model emerged from the of nucleotide incorporation rate of the polymerase obtained from literature [25]. This model was coded in MATLAB using SimBiology and Parallel Computing toolboxes. The program was run on a cluster having MATLAB version R2017a. We recommend having a cluster with minimum 50 GB RAM to run the program. The LAMP mechanism was divided into loop, series and parallel reactions which handle the SS, SL and PDS reactants, respectively, and they were named as SS loop, SL-PDS highway and PDS linker (Fig. 6) functions respectively. Each reaction function evaluates the reactant and generates the structures, number of copies and time of formation of the product. These details are then fed into the other reaction functions to generate products from the new reactants. The program has a main loop that goes through forty SS loop functions simultaneously followed by forty SL highway functions simultaneously followed by forty PDS functions simultaneously. As an example, consider a PDS product amplicon formed from an SS in the SS cycle and a cPDS product amplicon formed from a PDS in the SL-PDS highway. These product PDS amplicons are not evaluated in the SS cycle and SL-PDS highway functions. Instead, all the PDS product amplicons formed from SS and PDS amplicons are gathered and stored in a common PDS array. Then, forty PDS linker functions parse forty PDS cell arrays from this common PDS array. The program was run on a twenty-core cluster, each core able to run 2 computational threads parallelly. Hence, forty computational threads ran parallelly. Users having access to larger number of cores can modify the program to utilize the full potential of the available hardware. Each of these forty functions run on separate computational threads using the *parfor* function defined in the Parallel Computing toolbox. This allows to run the model for greater reaction times. Using these reaction constructs and concepts to determine the number of copies and time of formation of amplicons, LAMP reaction network was decentralized and solved in small chunks. However, the run times for this model were prohibitive beyond 14 minutes of reaction time for 20 starting copies of template and 9 minutes of reaction time for 200 starting copies of template because of the long run times of the program.

#### 4.2.3 Simulated gel

A matrix named *lastAmpliconsLen* contains the number of copies of each length of amplicon formed at the end of reaction time. The following steps were followed to create the simulated gel:

1. Amplicons that have lengths less than or equal to 1000 nucleotides are chosen from the *lastAmpliconsLen* matrix because the experimental gel ladder ranged from 100 to 1000 base pairs.
2. A MATLAB script was written to create the simulated gel image.
3. The ladder in the simulated gel was created by emulating the distances between the bands and the intensities of the bands in a ladder in experimental gels.
4. The bands in the adjacent lane were created by parsing the *lastAmpliconsLen* matrix and extracting the lengths of amplicons lying between 100 and 1000.
5. Each length is assigned a rectangle of appropriate width and height and is then positioned according to the ladder. The face color of the rectangles is set to white to show them as white bands against a black background.

## 5 Declarations of interest

None.

## CRediT authorship contribution statement

**Navjot Kaur:** Conceptualization, Methodology, Validation, Investigation, Writing - original draft, Visualization. **Nikhil Thota:** Methodology, Software, Data curation, Writing - original draft, Visualization. **Bhushan J. Toley:** Conceptualization, Methodology, Writing - review & editing, Supervision, Funding acquisition.

## Declaration of Competing Interest

The authors declare that they have no known competing financial interests or personal relationships that could have appeared to influence the work reported in this paper.

## Footnotes

# New abbreviations used in this manuscript: 1. SL (Single Loop) 2. T (Terminated) 3. PDS (Partially Double Stranded) 4. cPDS (child Partially Double Stranded) 5. SS (Single Stranded) 6. SSD (Single Stranded Dumbbell) 7. SPK (Stoichiometric and Pseudo Kinetic) 8. c-SPK (compressed Stoichiometric and Pseudo Kinetic) 9. PBE (Primer Based Extension) 10. CB (Color of Band).
