# Supplementary material for: A stoichiometric and pseudo kinetic model of loop mediated isothermal amplification
Source: Comput Struct Biotechnol J. 2020 Aug 31;18:2336–46. doi: 10.1016/j.csbj.2020.08.020 (PMC7493047; doi:10.1016/j.csbj.2020.08.020)
Supplement: Supplementary data 1 [file mmc1.pdf]

A stoichiometric and pseudo kinetic model of loop mediated isothermal amplification

Navjot Kaur[a†], Nikhil Thota[a,#,†], Bhushan J. Toley[a]*

[†]Equal contribution

[a] Department of Chemical Engineering
Indian Institute of Science
Bangalore, India
560012

NK: navjot@iisc.ac.in
NT: nikthota7@gmail.com
BT: bhushan@iisc.ac.in

*Correspondence to:
Bhushan J. Toley
Department of Chemical Engineering
Indian Institute of Science
C V Raman Avenue
Bangalore, India, 560012
Phone: +91-80-22933114

# Current address: Flat 101, Annapoorna Apartments,
Road 12, Banjara Hills,
Hyderabad, India
500034

Supplementary Figure S1. Publications on different isothermal DNA amplification techniques.

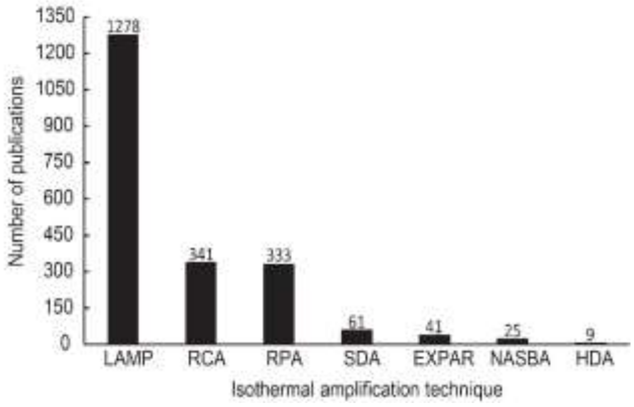

Supplementary Figure S1. Publications on different isothermal DNA amplification techniques. LAMP was found to be the most popular nucleic acid amplification technique in the research community for the last five years. Numbers as on 11th February 2020.

A literature search was conducted on Web of Science using the title of various isothermal amplification techniques as keywords and article as the document type filter. Names of the following isothermal amplification techniques were used as keywords: loop-mediated isothermal amplification (LAMP), rolling circle amplification (RCA), recombinase polymerase amplification (RPA), strand displacement amplification (SDA), exponential amplification reaction (EXPAR), nucleic acid sequence-based amplification (NASBA), and helicase dependent amplification (HDA). This revealed that over the last 5 years, LAMP has been the most frequently used technique for isothermal NAA with ~4x more publications compared to the second most popular technique.

Supplementary Figure S2. Illustration of all LAMP reaction pathways using amplicon structures for LAMP reaction with loop primers

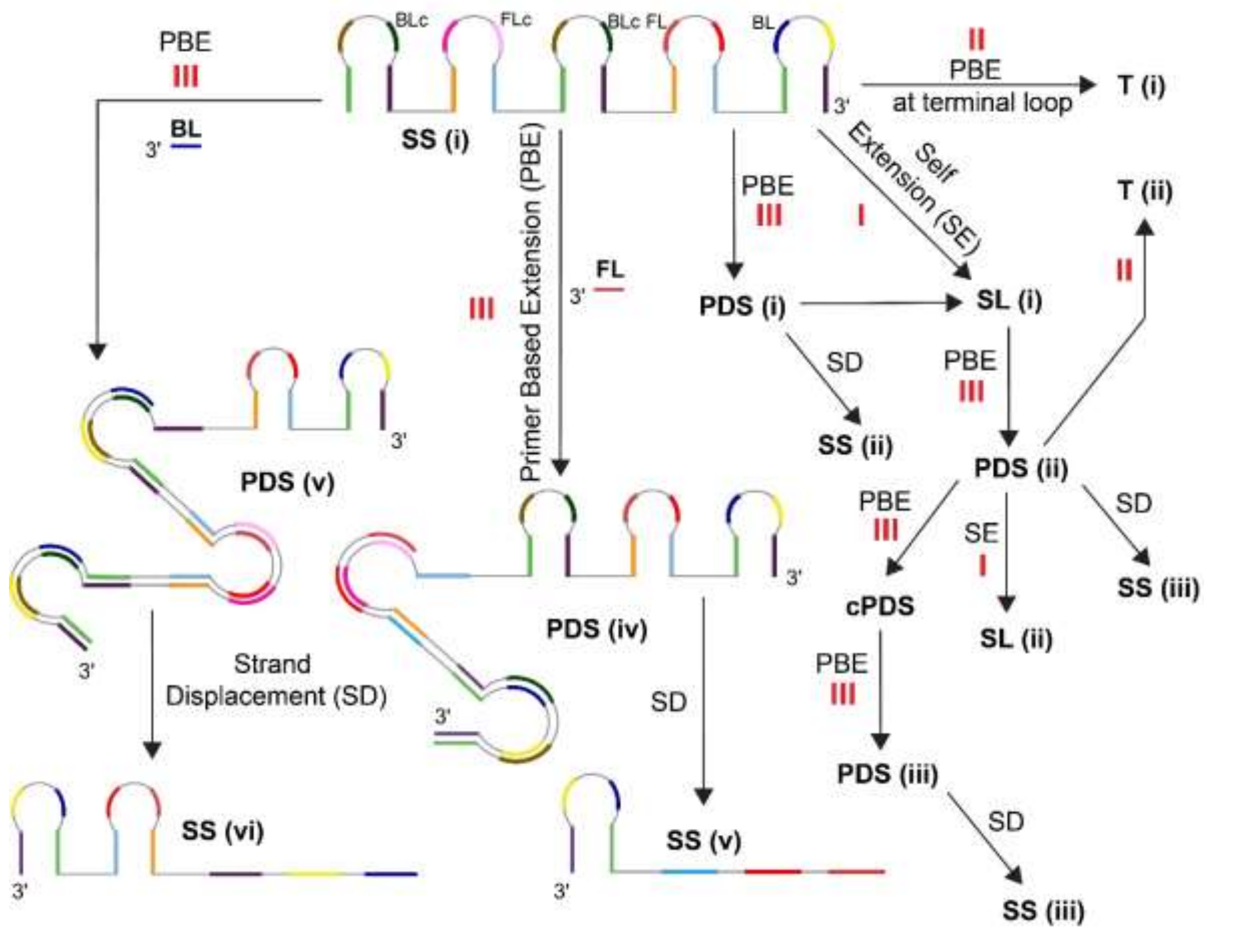

Supplementary Figure S2. Illustration of all LAMP reaction pathways using amplicon structures for LAMP reaction with loop primers. FL and BL represent forward and backward loop primer respectively. Roman numerals I, II, III, IV represent the different types of reactions each type of amplicon can undergo. I, II, III are as denoted in Figure 3 in main manuscript. Names of amplicons already present in Figure 3 have been maintained as is without showing their structures. Only structures of new amplicons generated due to addition of loop primers are shown.

LAMP reaction pathways in presence of loop primers along with four regular LAMP primers were studied to ensure that there exist only four types of LAMP amplicons. All amplicons formed in Figure 3 with only four primers are shown, marked with names and pathways for formation, as in Figure 3. Structures for new amplicons formed on addition of loop primers are shown. It was confirmed that the amplicons formed by loop primers fall only in the one of the four amplicon

types explained in the main manuscript. Since the loop primers FL and BL do not have complementary overhangs as in FIP and BIP, one patch of complementary sequence is missing at the 5’ end which inhibits loop formation at the 5’ end of amplicons. The three colored patches, shown as a horizontal line at 5’ end of SS(v) and SS(vi) represent these regions.

ESI Note S1 Brute force stoichiometric and pseudo kinetic (SPK) model

The first version of the SPK model was a brute force model that tracked each and every amplicon generated in LAMP. It determined and stored data for number of copies, length, sequence, formation time, parent amplicon and child amplicons for every amplicon. Formation time was based on the extension rate of the enzyme alone and had no dependence on concentrations of any species, i.e. assuming zeroth order kinetics. Using this program, the exact sequence of any species in any cell from this matrix can be extracted. All other assumptions made for developing the brute force model are provided in ESI Note S2. The model was programmed in MATLAB using the SimBiology toolbox and a detailed block diagram explaining flow of the computational program is provided in ESI Fig S2. Because this version of the model tracked each amplicon and stored the corresponding data, it required high computational power and data storage capacity. This led to a major roadblock as we couldn’t run the program of reaction time of more than two minutes.

Computational program for Brute force stoichiometric and pseudo kinetic model

This model is based on object-oriented methodology and individual class files were written for SS, SL and PDS reactants respectively. Objects are created using the constructor function and the class functions use these objects to create the product amplicon data. The product amplicon data along with the reactant data is stored in another set of objects and these objects are stored in an object array. There are separate object arrays for each reactant amplicon (SS, SL and PDS). After the

program has finished running, information about each amplicon produced in the reaction network can be accessed through the corresponding object array. The data in the object arrays is structured into two sections. The first section contains data for the parent of the reactant amplicon and the reactant amplicon's formation time, length, sequence and the number of copies. The second section contains information about the sequence of the product amplicon, the nucleotides used by the reactant amplicon to form the product and the number of copies of the product. The program only requires sequence of the dumbbell to initiate the calculations and it builds the entire reaction network independently.

An important point to note is that the program calculates the reaction network for only one dumbbell produced from the target strand. The program is first run for the sense strand dumbbell and then for the anti-sense strand dumbbell to cover the entire reaction network. The brute force model builds the reaction network generation by generation. The current generation is defined as a row vector which contains the reactant amplicons and the next generation is another row vector which contains the products of the reactants. The program goes through each entry in the current generation and the products formed from each entry are stored in the next generation amplicon array. After it goes through each entry in the current generation, it then appends the next generation to the bottom of the reaction network matrix and makes it the current generation, repeating the whole process. The reaction network keeps expanding until formation time of amplicons is greater than reaction time. The brute force model analyzed each amplicon individually, determined and stored its number of copies, length, sequence, formation time, parent amplicon and child amplicons. A detailed block diagram explaining the computational program flow is provided below:

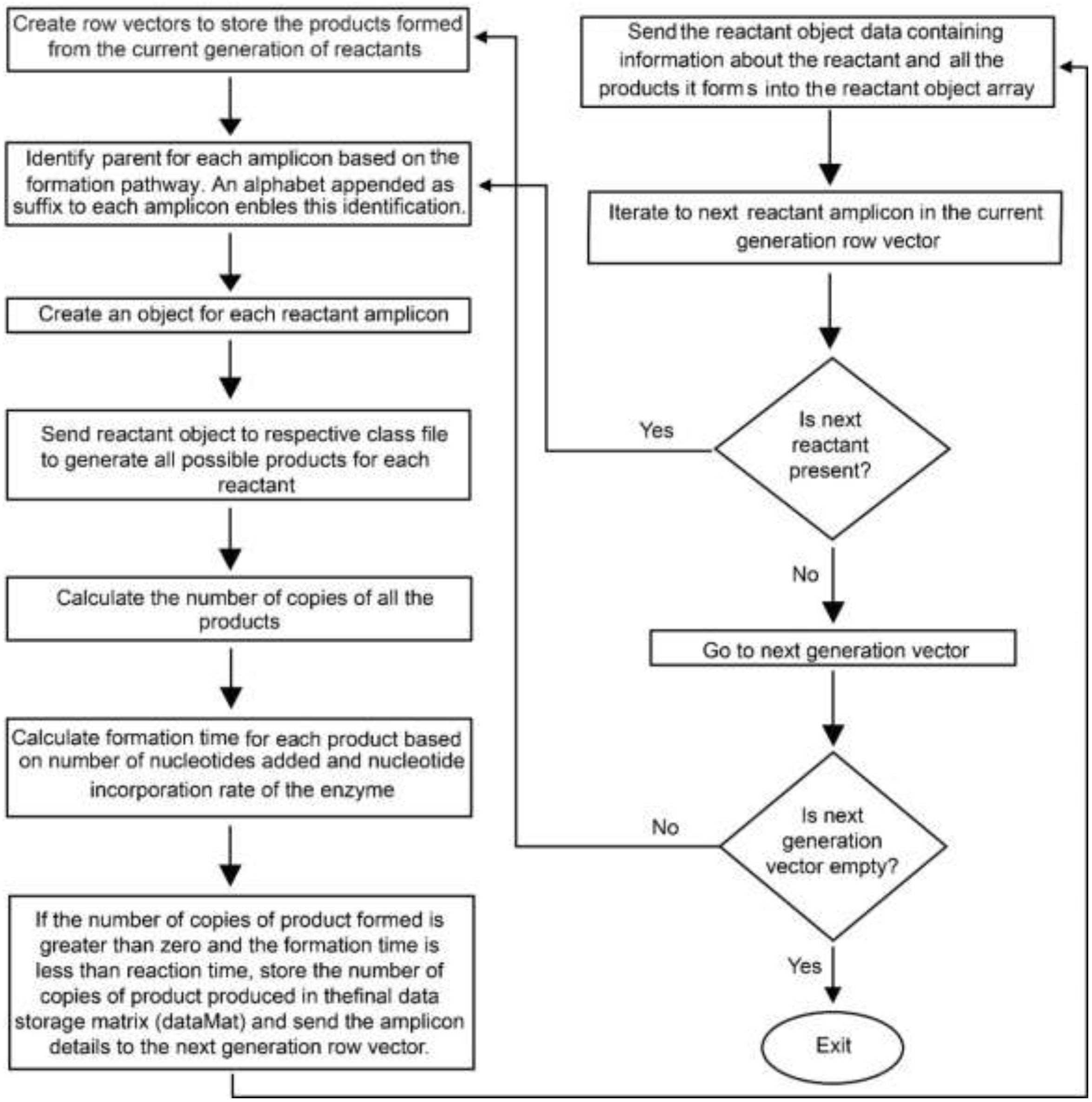

Supplementary Figure S3. Block diagram for the logic used for creating brute force stoichiometric and pseudo-kinetic model.

ESI Note S2. Assumptions for developing Brute force and compressed stoichiometric and pseudo kinetic (SPK) model

1. The concentration of nucleotides and primers remains constant throughout the reaction.
2. Time taken to form a product amplicon from a reactant amplicon is taken to depend only on the extension time obtained from the nucleotide incorporation rate of the enzyme. This

assumption is based on the fact that the time scale for primer annealing and enzyme attachment to template is of the order of nanoseconds while the extension time is of the order of seconds.

3. Probability of formation of different amplicon types is constant with respect to reaction time.
4. Primers are equally likely to anneal at all primer annealing sites present in an amplicon. This implies that there is equal probability of formation of partially double stranded (PDS) and terminated (T) amplicons from a single-stranded (SS) or PDS amplicon.
5. Once formed, T amplicons do not participate in any further reactions.
6. Nucleotide incorporation rate of the enzyme (Bst 2.0) is taken to be constant for entire duration of the reaction. In reality, nucleotide incorporation rate should decrease over time owing to decreasing concentrations of primers and nucleotides in the reaction mix and increasing length of the amplicons.
7. Formation time of an SS amplicon formed from a PDS amplicon via different reaction pathways is taken to be the same. Furthermore, SS formation time is taken to be equal to the time it takes to generate a single-loop (SL) amplicon from a PDS.

   Consider a PDS amplicon (Fig S4, PDS) to which an FIP (5'F1c –F2) can anneal at F2c loop to produce a child PDS (cPDS) and a BIP (5' B1c-B2) can anneal at the terminal B2c loop to produce T. The 3' end of the single-stranded section of PDS amplicon can also self-anneal to produce an SL. In all these cases an SS is formed via strand displacement, but the time taken for strand displacement is different for each case. It depends on number of nucleotides to be added to form the corresponding products. Time taken for strand displacement of SS for different product types varies as: formation time for T > formation

time for SL > formation time for child PDS. If there were 'n' primer annealing locations on a PDS which could lead to formation of child PDS and T, there would be an SS displaced at 'n' different times and the program would require 'n' separate variables to store SS formation times. This increases the memory space requirement of the program and slows down its execution speed. SL formation pathway was chosen to define SS formation time because we found that probability of formation of an SL amplicon is considerably larger than that for T and child PDS. This can be explained from the fact that the first step in SL formation reaction is enzyme binding while for child PDS and T it is primer annealing. Since the forward binding constant for enzyme attachment is two orders of magnitude greater than forward binding constant for primer annealing, a larger fraction of reactant copies is transformed to SL.

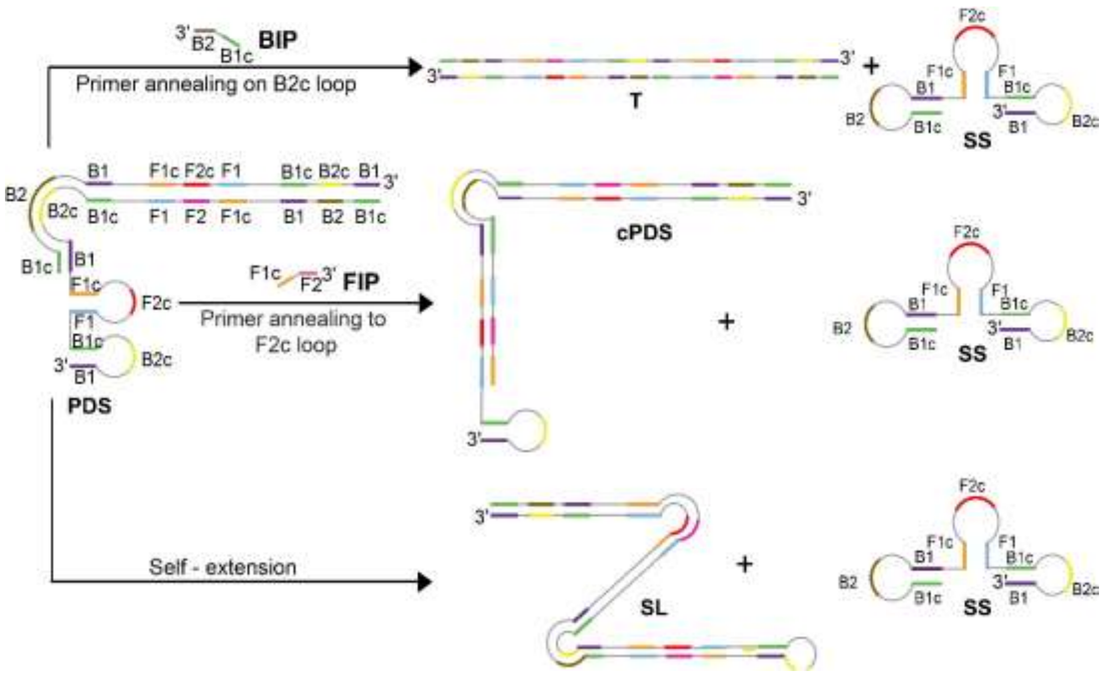

Supplementary Figure S4. Formation time of single-stranded (SS) amplicon.

SS amplicons can be formed by strand displacement when a partially double stranded (PDS) amplicon forms a terminated (T), child PDS or single-loop (SL) amplicon. The number of nucleotides incorporated for the three pathways is different, leading to different formation times for the same length SS amplicon.

8. All amplicons are assumed to be rigid molecules such that they do not coil up to have a shorter length than their actual length.
9. The double-stranded segments of different amplicon types are considered not to breathe as that would generate even greater number of primer annealing sites and increase the complexity of the model.
10. For determination of the last amplicon, the formation time of the product is compared with the total reaction time. If the formation time of the product exceeds the reaction time, then the corresponding reactant has to be the last amplicon. In case of SL reacting to form PDS it is easy to determine if SL is the last reactant because an SL can produce only a PDS amplicon. But in cases like SS and PDS where parallel reactions occur producing multiple products, only the SL product amplicon is considered to determine if the last amplicon is a SS or PDS reactant. Since majority of the SS and PDS reactants are converted to SL and because it takes the longest time to generate, if formation time for SL exceeds reaction time, we consider the SS or PDS to be the last amplicon.
11. PDS products produced from an SS and child PDS product produced from a PDS do not form any further PDS amplicons in the c-SPK model. For these PDS product amplicons produced from an SS or PDS to further form PDS amplicons, primer annealing is required and as explained in point 7, self-extension is more favorable than primer annealing. We

also did theoretical calculations and found that the number of amplicons formed via the two abovementioned pathways would be negligible.

12. The extension process was assumed to be a single step process in the c-SPK model. Extension proceeds by incorporation of oligomers having length equal to the number of nucleotides to be added to the reactant for each extension step.

ESI Note S3. Length of different LAMP amplicon types

It was observed that the structure of dumbbell and following all LAMP amplicons only comprised of F1, F2, B1, B2 and their complementary patches. To make the representation of sequences simpler, the repeating patches F1c-F2c-F1 and F1c-F2-F1 are represented by F patches (Fig. S5A), and patches B1c-B2c-B1 and B1c-B2-B1 are represented by B patches (Fig. S5A). It is these 'F' and 'B' patches which are repeatedly added to newly forming amplicons in the exponential phase of LAMP reaction. The nucleotides present between the 'F' and 'B' patches are represented by 'D' patches (Fig. S5A). The regions that form F and B patches are obtained from the target DNA sequence and the number of nucleotides in each patch can be calculated from the sequence of the target DNA. Fig. S5B shows the number of nucleotides present in the target sequence considered for our model and the total number of nucleotides present in the F, B and D patches in the dumbbell formed from this target are calculated as follows:

$$Number\ of\ nucleotides\ in\ B\ patch\ =\ 22\ +\ 23\ +\ 9+\ 19\ +\ 22\ =\ 95$$

$$Number\ of\ nucleotides\ in\ F\ patch\ =\ 19\ +\ 19\ +\ 20\ +\ 1\ +\ 19\ =\ 78$$

$$Number\ of\ nucleotides\ in\ D\ patch\ =\ 33$$

$$Total\ number\ of\ nucleotides\ in\ dumbell\ =\ 206$$

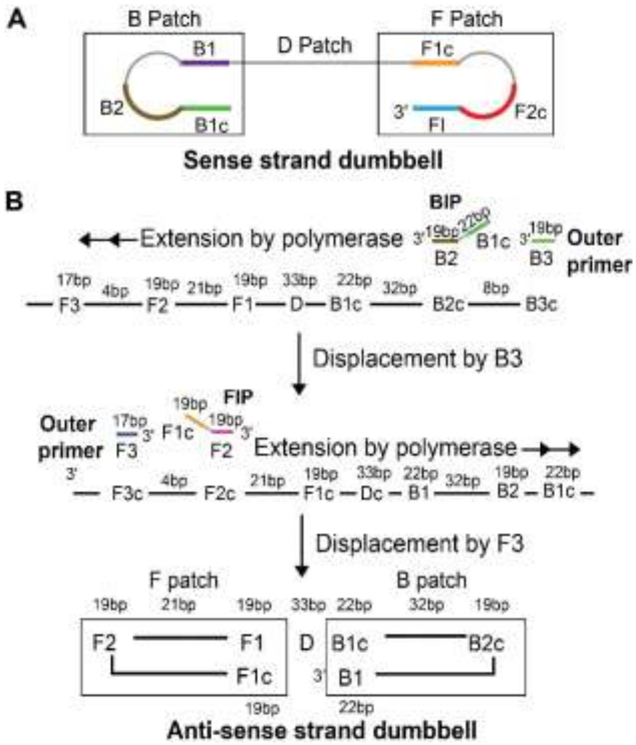

Supplementary Figure S5. Calculation of number of nucleotides from target sequence. (A) Sense strand dumbbell (B) Calculation of number of nucleotides in 'F', 'B' and 'D' patches.

Since the compressed stoichiometric and pseudo kinetic (c-SPK) model is developed using MATLAB, we came up with a matrix-based representation for length of different types of amplicons (SS, SL, PDS and T) generated in LAMP. These matrices, called patch matrices, store sequence of patches present in the amplicons. The patch matrices are utilized to identify primer annealing sites present on amplicons and to calculate length of amplicons and number of nucleotides added to a reactant to form corresponding product. The D patch does not possess sites for primer annealing and hence is not stored in patch matrices. The patch matrices for SS (Fig. S6A) and SL amplicons (Fig. S6B) are denoted by ss (Fig. S6A) and ds (Fig. S6B) respectively. The PDS structure (Fig. S6C) is divided into 3 parts and each part is stored in different patch matrices; namely es, ls and ss. The ss patch matrix of PDS stores the sequence of the SS amplicon

that will be generated by strand displacement when a PDS undergoes self-extension or primer annealing. The es patch matrix of PDS stores the strand of PDS that is annealed to ss as well as the part containing single loops where primer can anneal and form child PDS or T. The ls patch matrix stores only the single-loop region sequence where a primer can anneal to form a child PDS or T. For all patch matrices except ds, the 3' end is located at the end of the matrix whereas for 'ds' patch matrix it is located at the beginning of the second row (Fig. S6 B).

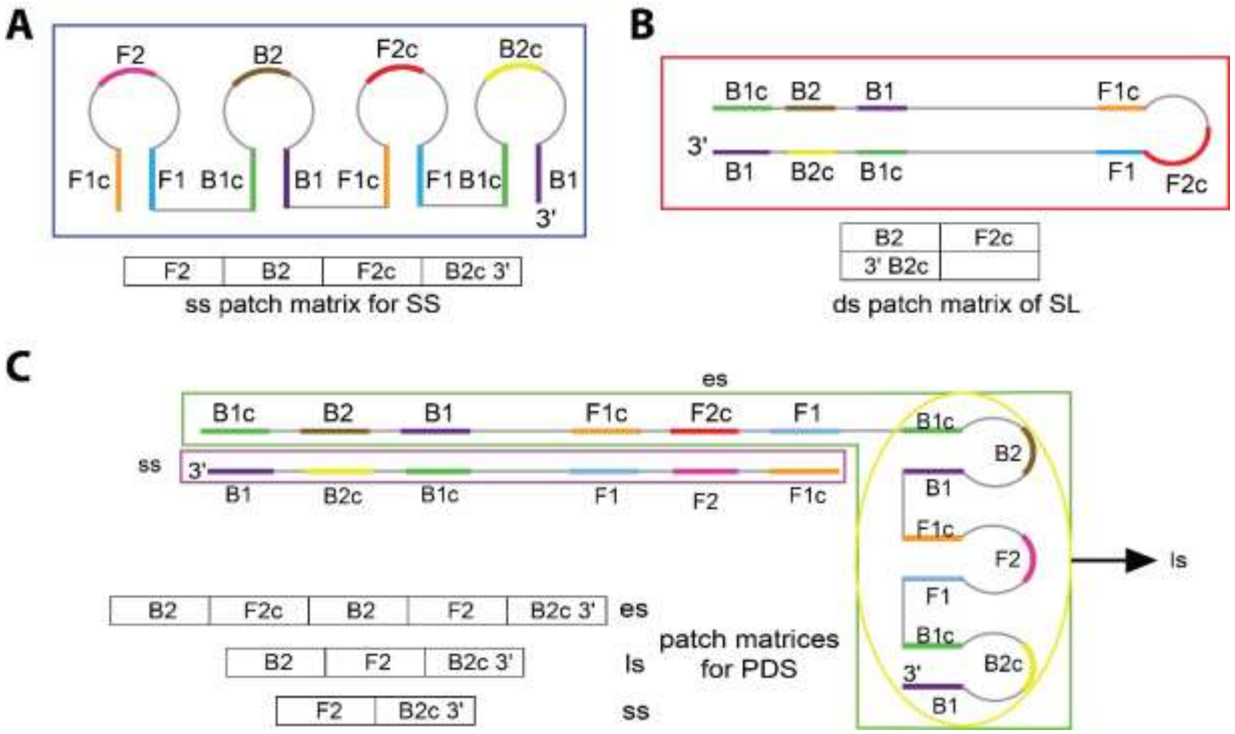

Supplementary Figure S6. Matrix based representation for calculating length of different categories of LAMP amplicons. (A) SS amplicon (B) SL amplicon and (C) PDS amplicon

The program calculates number of columns present in patch matrices by using the length function inbuilt in MATLAB and that is defined as the length of a patch matrix. Length of an SS, SL and PDS amplicon is represented by length of ss, ds and es patch matrix, respectively. Length of T amplicon is calculated using length of the SS or PDS reactant which formed the T amplicon,

because to form a T amplicon one has to create a complement of the entire ss length in case of SS reactant or the entire es length in case of PDS amplicon. After analyzing the trend in patch sequences, we found that if length of patch matrix is even then there should be an equal number of ‘B’ and ‘F’ patches and if length is odd then there should be an excess of either ‘B’ or ‘F’ patch depending on whether the end of the patch matrix has a ‘B’ or ‘F’ patch, respectively. The formulae used for calculating the number of ‘B’,’F’ and ‘D’ patches in the patch matrix are defined below. Multiplying the number of patches with the number of nucleotides present in each patch provides the total number of nucleotides present in an amplicon.

Case I: Length of patch matrix is even:

$$\text{Number of}'\text{B}'\text{patches in patch matrix} = \frac{\text{length of patch matrix}}{2} \qquad - \text{Eqn 1}$$

$$\text{Number of}'\text{F}'\text{patches in patch matrix} = \frac{\text{length of patch matrix}}{2} \qquad - \text{Eqn 2}$$

$$\text{Number of}'\text{D}'\text{patches in the patch matrix} = (\text{Eqn 1}) + (\text{Eqn 2}) - 1 \qquad - \text{Eqn 3}$$

The total number of nucleotides in the amplicon, equivalent to length of the amplicon is calculated as follows:

$$\text{Length} = (Eqn\ 1) * (B\ patch\ nucleotides) + (Eqn\ 2) * (\text{F patch nucleotides}) + (Eqn\ 3) * (D\ patch\ nucleotides)$$

Case II: Length of patch matrix is odd:

(a) Length of patch matrix is odd and 3’ end has a ‘B’ patch

$$\text{Number of}'\text{B}'\text{patches in the patch matrix} = \text{ceiling}\left(\frac{\text{length of patch matrix}}{2}\right) \qquad - \text{Eqn 4}$$

$$\text{Number of}'\text{F}'\text{patches in the patch matrix} = (\text{length of patch matrix}) - (\text{Eqn 4}) \qquad - \text{Eqn 5}$$

$$\text{Number of}'\text{D}'\text{patches in the patch matrix} = (\text{Eqn 4}) + (\text{Eqn 5}) - 1 \qquad - \text{Eqn 6}$$

The total number of nucleotides in the amplicon, equivalent to length of the amplicon is calculated as follows:

$$\text{Length} = (Eqn\ 4) * (B\ patch\ nucleotides) + (Eqn\ 5) * (\text{F patch nucleotides}) + (Eqn\ 6) * (D\ patch\ nucleotides)$$

(b) Length of patch matrix is odd and 3’ end has an ‘F’ patch

$$\text{Number of}'\text{F}'\text{patches in the patch matrix } = \text{ceiling}\left(\frac{\text{length of patch matrix}}{2}\right) \quad - Eqn\ 7$$

$$\text{Number of}'\text{B}'\text{patches in the patch matrix } = (\text{ length of patch matrix}) - (\text{Eqn } 7) \quad - Eqn\ 8$$

$$\text{Number of}'\text{D}'\text{patches in the patch matrix } = (\text{Eqn } 7) + (\text{Eqn } 8) - 1 \quad - \text{Eqn } 9$$

The total number of nucleotides in the amplicon, equivalent to length of the amplicon is calculated as follows:

$$\text{Length} = (Eqn\ 8) * (B\ patch\ nucleotides) + (Eqn\ 7) * (\text{F patch nucleotides}) + (Eqn\ 9) * (D\ patch\ nucleotides)$$

ESI Note S4. Calculation of number of nucleotides added to form new amplicons in LAMP

(i) Number of nucleotides added to SL to form PDS

Fig. S7 demonstrates the formation of a PDS amplicon from SL. The number of ‘B’ and ‘F’ patches added can be calculated using the length of the ds patch matrix of reactant SL. The number of nucleotides added to an SL to form PDS can be calculated by multiplying the number of patches with the number of nucleotides in each patch.

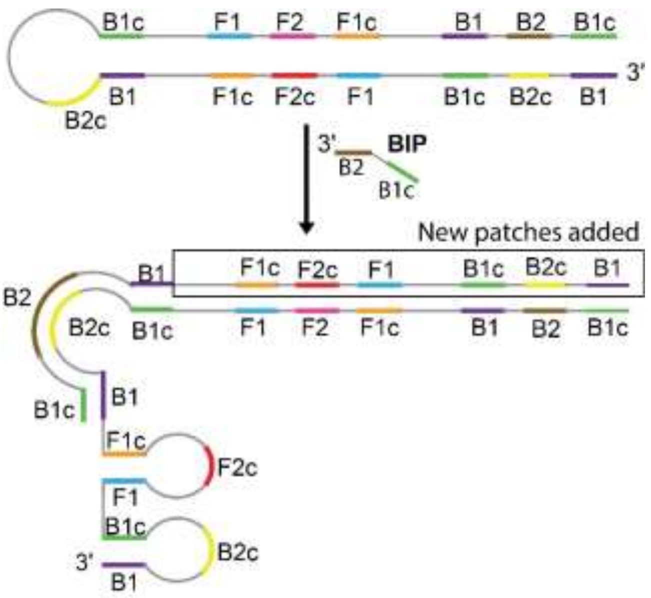

Supplementary Figure S7. Formation of a PDS amplicon from an SL amplicon.

(ii) Number of nucleotides added to SS to form T

Fig. S8A demonstrates formation of T from SS via primer annealing and extension. The same number of 'B', 'F' and 'D' patches, as present in ss patch matrix, are added to SS to form T amplicon. The number of nucleotides added to an SS to form T can be calculated by multiplying the number of patches with the number of nucleotides in each patch.

(iii) Number of nucleotides added to SS to form SL

Fig. S8B demonstrates formation of SL from SS via self-extension. The following set of equations describe the calculations done for finding the number of nucleotides added to an SS to from SL, depending on the nature of sequence of parent SS.

If the 3' end of SS is a 'B' patch:

$$\text{B patches added} = (\text{No. of B patches in } SS) - 1 \qquad - Eqn\ 10$$

$$\text{F patches added} = (\text{No. of F patches in SS}) \qquad - Eqn\ 11$$

$$\text{D patches added} = (\text{No.} \, of \text{ B patches in SS } + \text{No. of F patches in } SS) - 1 \quad - \text{ Eqn 12}$$

If the 3’ end of SS is an ‘F’ patch:

$$\text{B patches added} = (\text{No. of B patches inSS}) \quad - Eqn\ 13$$

$$\text{F patches added} = (\text{No. of F patches in SS}) - 1 \quad - Eqn\ 14$$

$$\text{D patches added} = (\text{No. of B patches in SS } + \text{No. of F patches in SS}) - 1 \quad - \text{ Eqn 15}$$

(iv) Number of nucleotides added to SS to form PDS

Fig. S8C demonstrates formation of PDS from SS via primer-annealing and extension. The location(s) of primer binding sites (F2c or B2c) are determined for the parent SS amplicon using the find function in MATLAB. The primer binding site at the 3’ end is not considered as it leads to formation of a T amplicon and not PDS. As shown in Fig. S8C, if the primer anneals to the second loop (F2c) from right of the SS amplicon, then the length of the SS patch matrix of PDS product formed gives the number of patches added to SS to form PDS. If the number of patches in the ss matrix of PDS product is even, then there will be an equal number of ‘B’ and ‘F’ patches added. However, if the number of patches is odd then there will be an excess of either ‘B’ or ‘F’ patches added depending on location of primer annealing. This analysis is repeated for each of the primer annealing sites on the SS amplicon.

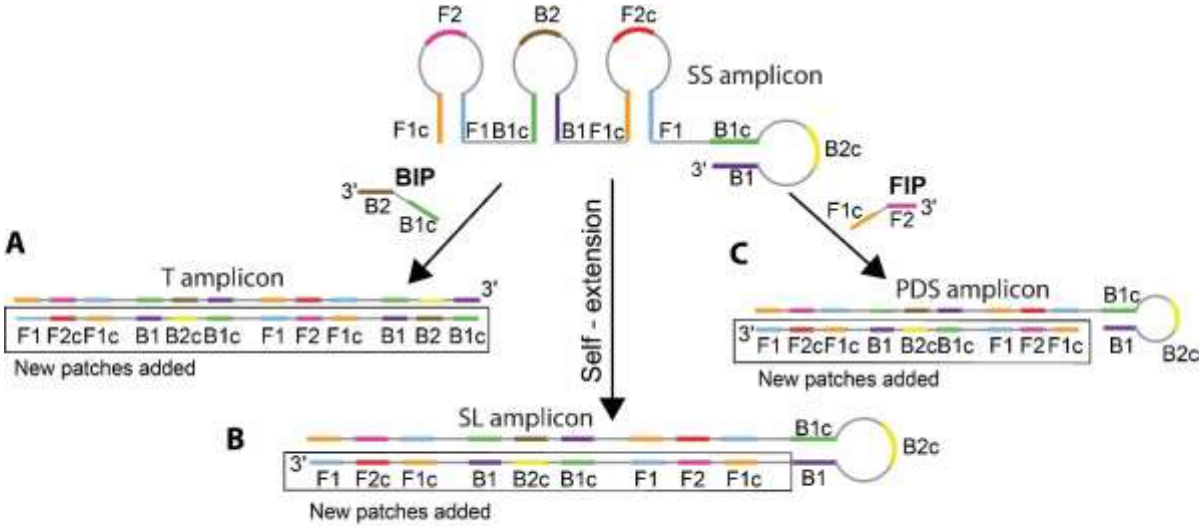

Supplementary Figure S8. Formation of different amplicon types from SS amplicon. (A) Formation of T from SS (B) Formation of SL from SS (C) Formation of PDS from SS

(v) Number of nucleotides added to PDS to form T

Fig. S9A demonstrates formation of T from PDS via primer annealing and extension. The new strand generated to create the T amplicon encompasses the entire es strand of the PDS amplicons. The number of 'B' and 'F' patches in T will be same as in the es patch matrix of PDS. The number of D patches can be calculated accordingly.

(vi) Number of nucleotides added to PDS to form SL

Fig. S9B demonstrates formation of SL from PDS via self-extension. The following set of equations describe the calculations done for finding the number of nucleotides added to a PDS to form SL, depending on the nature of sequence of parent PDS:

If the 3' end of es strand is a 'B' patch:

$$\text{B patches added} = (\text{No. of B patches in es of PDS}) - 1 \qquad - Eqn\ 16$$

$$\text{F patches added} = (\text{No. of F patches in es of PDS}) \qquad - Eqn\ 17$$

$$\text{D patches added} = (\text{ No. B patches in es ofPDS} + \text{No. F patches in es ofPDS }) - 1 - \text{Eqn 18}$$

If the 3’ end of es strand is an F patch then,

$$\text{B patches added} = (\text{No. of B patches in es of PDS}) \qquad - Eqn\ 19$$

$$\text{F patches added} = (\text{No. of F patches in es of PDS}) - 1 \qquad - Eqn\ 20$$

$$\text{D patches added} = (\text{No. B patches in es ofPDS} + \text{No. F patches in es ofPDS }) - 1 - \text{Eqn 21}$$

(vii) Number of nucleotides added to PDS to form child PDS

A child PDS is the product PDS amplicon produced from a parent PDS amplicon. Fig. S9C demonstrates formation of child PDS from parent PDS via primer annealing and extension. Length of the ss patch matrix of child PDS product gives the number of patches added to form the child PDS. The location(s) of primer binding sites (F2c or B2c) are determined for the ls patch matrix of PDS amplicon using the find function in MATLAB. The primer binding site at the 3’ end is not considered as it leads to formation of a T amplicon and not child PDS. As shown in Fig. S9C, if the primer anneals to the second loop (F2c) from right of the PDS amplicon, then the length of the SS patch matrix of child PDS product formed gives the number of patches added to parent PDS to form child PDS amplicon. If the length of ss patch matrix of child PDS is even, then there will be equal number of ‘B’ and ‘F’ patches added. If the length of ss patch matrix of child PDS is odd, then there will be an excess of either ‘B’ or ‘F’ patches added depending on the location of primer annealing.

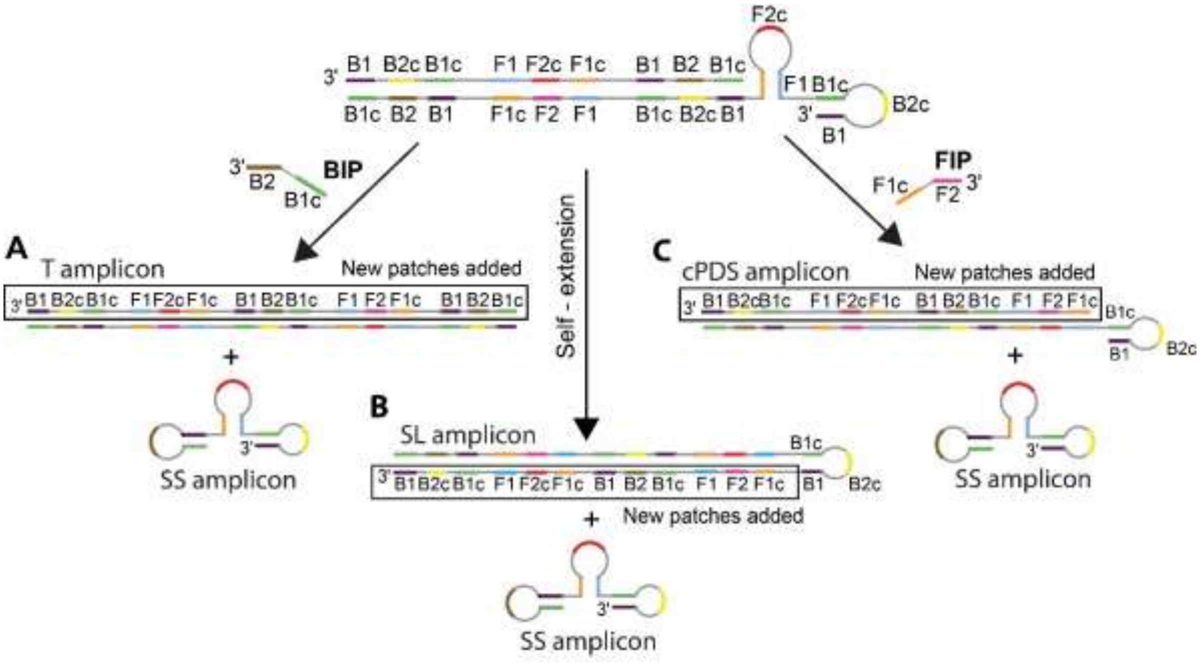

Supplementary Figure S9. Formation of different amplicon types from PDS amplicon. (A) Formation of T from PDS, (B) Formation of SL from PDS, and (C) Formation of child PDS (cPDS) from PDS.

ESI Note S5. Formulae for calculating the number of copies of product formed from a reactant

(i) Formation of SL from SS

$$\text{Number of copies of SL} = \text{round}((\text{probSL}) * (\text{number of copies of SS}))$$

(ii) Formation of PDS and T from SS

Consider 'n' primer annealing sites available on SS out of which n-1 can form 'n – 1' different types of PDS amplicons and the terminal primer annealing site located at the 3' end forms a T amplicon.

$$\text{Number of copies of T and each PDS formed from an SS}$$
$$= \text{round}\left(\frac{\text{number of copies of SS} - \text{number of copies of SL formed from SS}}{\text{number of primer annealing sites}}\right)$$

(iii) Formation of SS from PDS

$$\text{Number of copies of SS} = \text{number of copies of PDS}$$

(iv) Formation of SL from PDS

$$\text{Number of copies of SL} = \text{round}((\text{probSL}) * (\text{number of copies of PDS}))$$

(v) Formation of T and child PDS from PDS

Consider ‘n’ primer annealing sites available on PDS out of which n-1 can form ‘n – 1’ different types of child PDS and the terminal primer annealing site located at the 3’ end forms a T amplicon.

$$\text{Number of copies of T and each child PDS formed from PDS}$$

$$= \text{round}\left(\frac{\text{number of copies of SS} - \text{number of copies of SL formed from PDS}}{\text{number of primer annealing sites}}\right)$$

Supplementary Figure S10. Block diagram for SSD and SS cycles

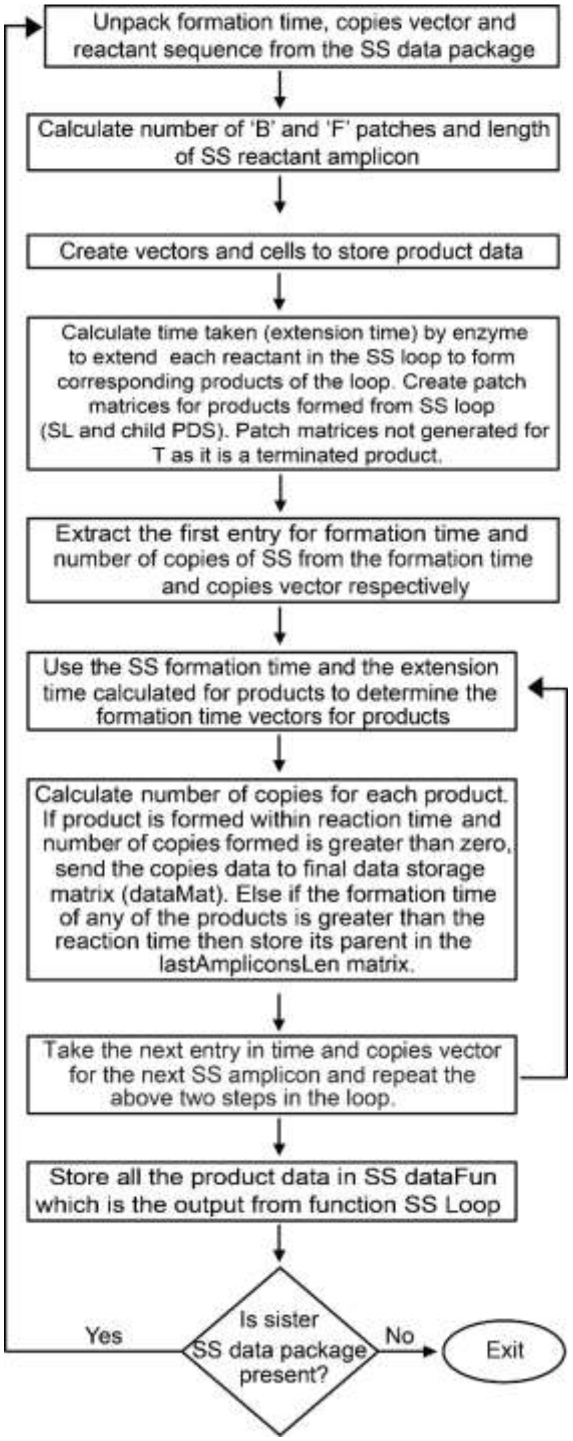

Supplementary Figure S10. Block diagram representing computational program flow for SSD and SS cycle.

Supplementary Figure S11. Block diagram for SL-PDS highway

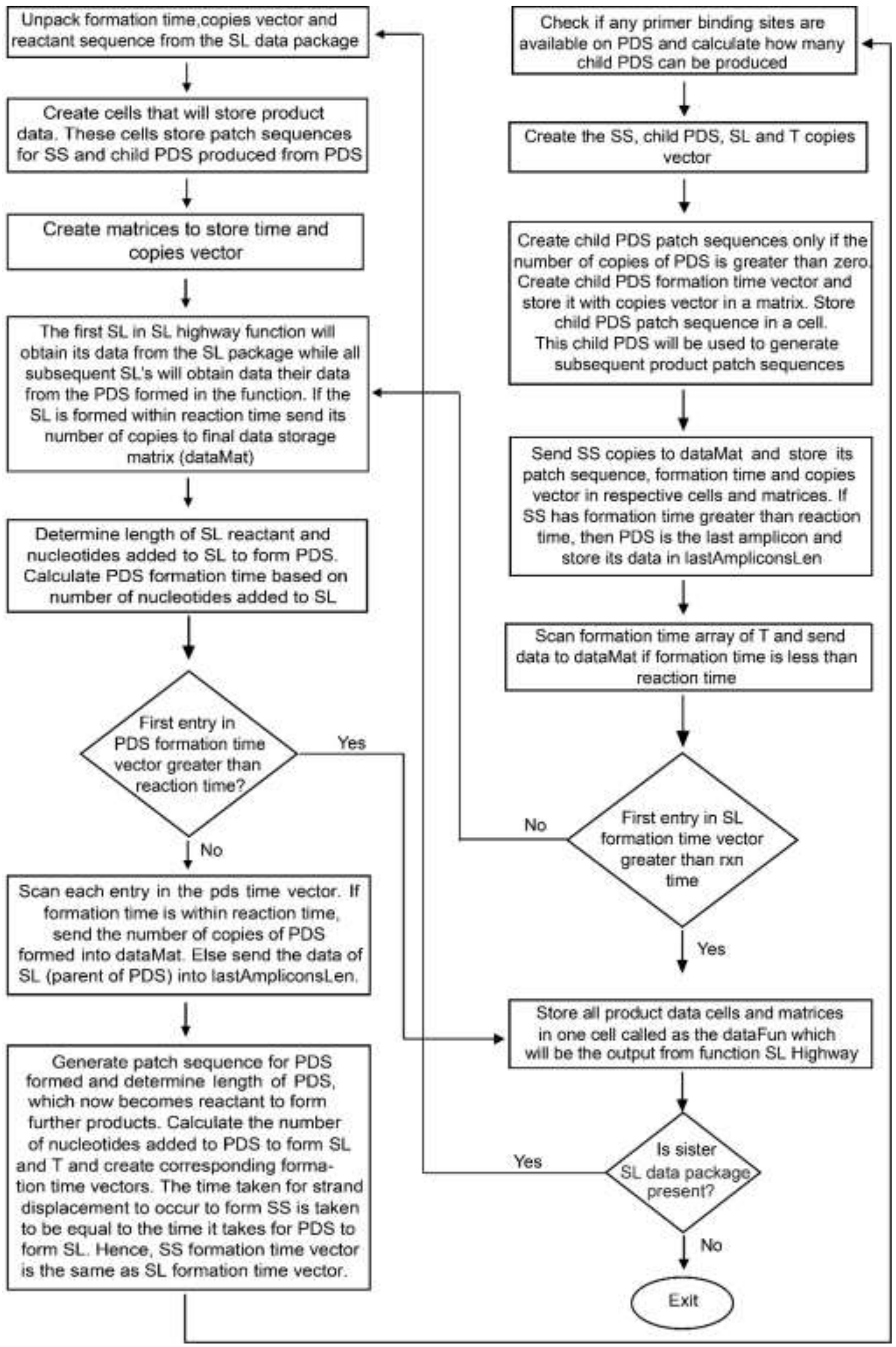

Supplementary Figure S11. Block diagram representing computational program flow for SL-PDS highway.

Supplementary Figure S12. Block diagram for PDS linker

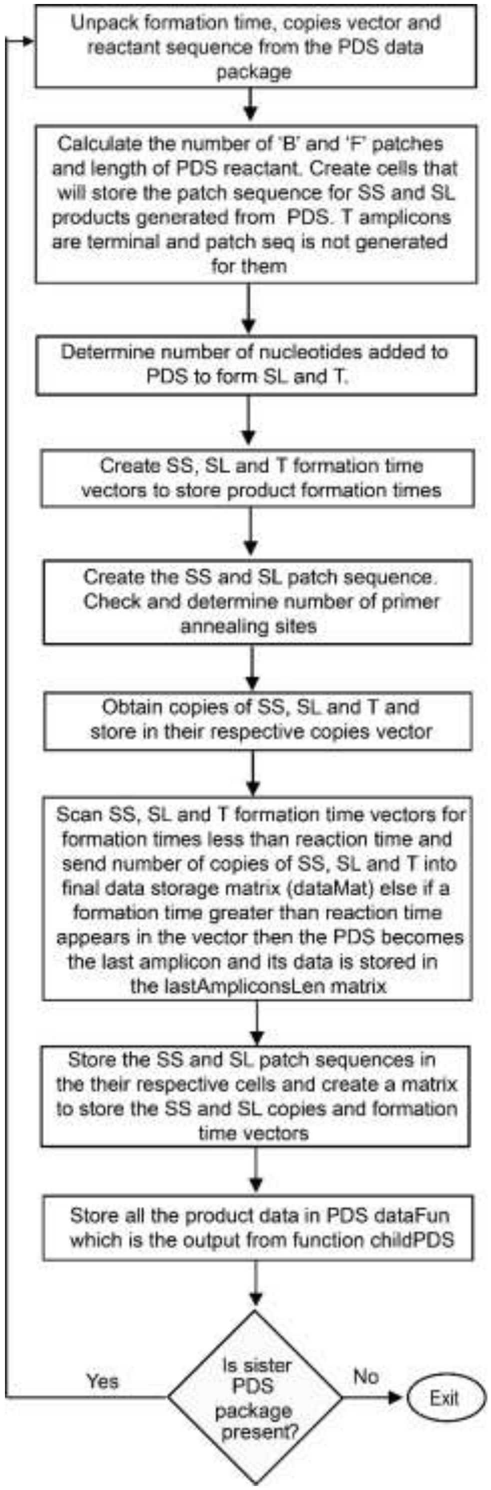

Supplementary Figure 12. Block diagram representing computational program flow for PDS linker.

Supplementary Figure S13 Estimation of reactant concentrations to solve kinetic model for converting enzyme units into molarity

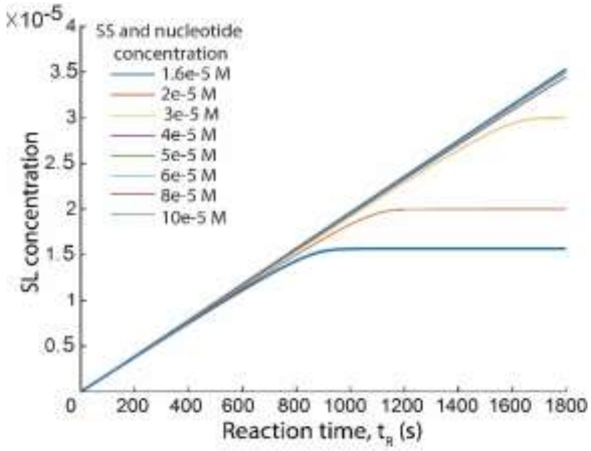

Supplementary Figure S13. Estimation of reactant concentrations to solve kinetic model for converting enzyme units into molarity
Concentration of SS and nucleotides was iterated to find their minimum concentration above which only the enzyme concentration effected the reaction kinetics.

Enzyme concentrations are conventionally reported in units/ml while we required enzyme concentrations in molarity to solve the kinetic model for calculating probability of formation of different types of LAMP amplicons. In order to solve the kinetic model for conversion of enzyme units into molarity, the starting concentration of the reactants (SS and nucleotides) was estimated such that reaction is only sensitive to the enzyme concentration. This was done by choosing a relatively high enzyme concentration (70 nM) and steadily increasing the concentrations of both SS and nucleotides. The color-coded curves in Fig. S16 correspond to different SS and N concentrations, varying from 1.6 x 10^{-5}M to 10 x 10^{-5}M. We found that concentration of SS and N above 4x10^{-5}M resulted in negligible change in the rate of generation of SL. Hence, starting concentration of SS and N was taken as 10 x 10^{-5} M.

Supplementary Figure S14 Converting enzyme units into molarity

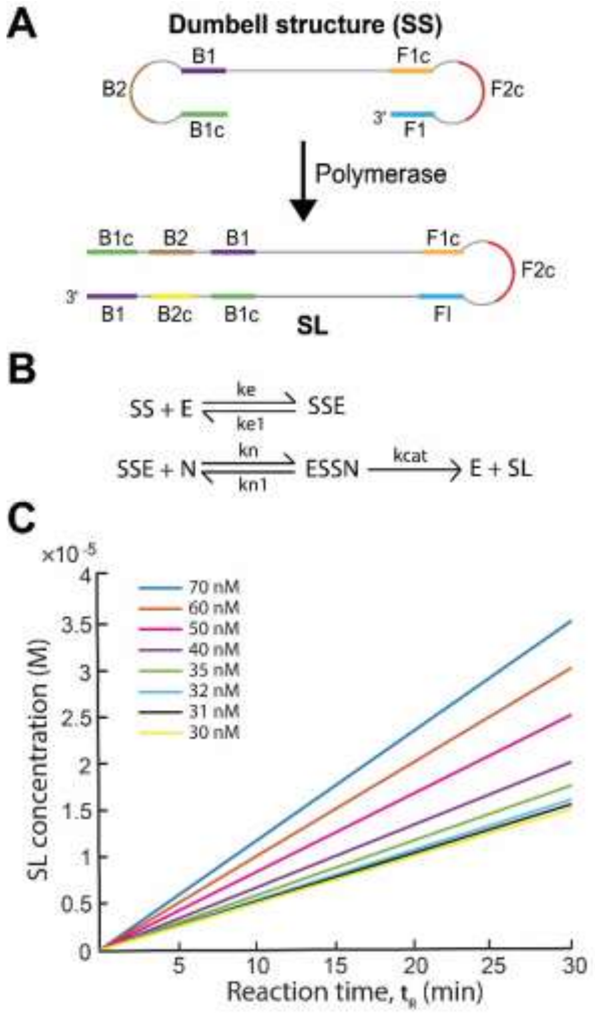

Supplementary Figure S14. Conversion of enzyme units into molarity. (A) Schematic for conversion of SS into SL. (B) Set of equations used for solving the kinetic model. (C) Results for iteration of enzyme concentration. The kinetic model was solved for a reaction time of 30 minutes with initial concentration of SS and nucleotides equal to 10^{-4} M each. E – enzyme, N – nucleotides, ke and ke1 – enzyme binding and unbinding rate constants, kn and kn1 – nucleotide binding and unbinding rate constant and kcat - catalytic rate constant of the enzyme.

In order to be consistent with the concentration units of different species involved in LAMP reactions, the conventional representation of enzyme concentration in enzyme units was converted into molarity. This kinetic model was created using the Simbiology toolbox of MATLAB. The product catalogue for Bst 2.0 (M0538S, NEB) specifies that one unit of the enzyme is defined as the amount that incorporates 25nmoles of dNTPs in 30 minutes at 65°C. For the considered amplification from SS to SL, it requires addition of 128 nucleotides to form the product. It was assumed that all dntps are incorporated in a single extension step and the species dntps was taken to be a collection of 128-mers. As explained in the previous section, the concentration of SS reactant amplicons and nucleotides was kept in excess to ensure that only the enzyme concentration impacted reaction kinetics. A range of enzyme concentration (70nM to 30nM) was iterated to find the enzyme concentration that enabled conversion of reactant equivalent to the activity of one unit of the enzyme. One unit of enzyme (Bst 2.0, NEB) incorporates 25nmoles of dNTPs in 30 minutes at 65°C. Ode15s solver was used for solving the differential equations corresponding to the above set of reactions. List of parameters required to solve this kinetic model is provided in ESI Table S3. It was found that 31nM of the enzyme could incorporate the same number of nucleotides as 1 unit of enzyme under the reaction conditions as specified by the enzyme manufacturer

Supplementary Table S3. List of the parameters used for converting enzyme units into molarity

S. No.	Parameter name	Value	Unit
1	Volume	12.5E-6	L
2	Enzyme binding rate constant (ke)	1E7	Molarity^{-1} s^{-1}
3	Enzyme unbinding rate constant (ke1)	1E-5	s^{-1}
4	Nucleotide binding rate constant (kn)	5E5	Molarity^{-1} s^{-1}
5	Nucleotide unbinding rate constant (kn1)	1E-4	s^{-1}

Supplementary Figure S15. Probability of formation of different types of LAMP amplicons

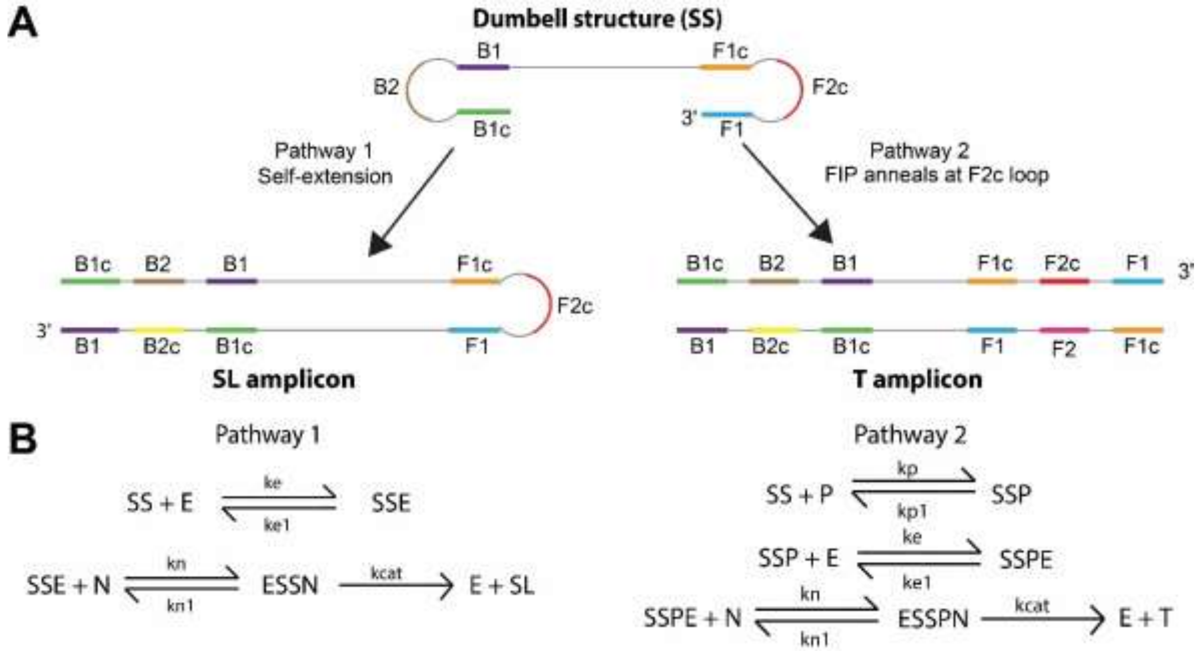

Supplementary Figure S15. Probability of occurrence of self-extension and primer-based extension for nucleic acid amplification in LAMP. (A) Schematic for the two possible pathways of self-extension and primer annealing. (B) Set of equations solved for the kinetic model for calculating the probabilities. The probability for following pathway 1 was found to be 0.674 and 0.326 for pathway 2. E – enzyme, N – nucleotides, P - primer, ke and ke1 – enzyme binding and unbinding rate constants, kn and kn1 – nucleotide binding and unbinding rate constant, kp and kp1 – primer binding and unbinding rate constant, and kcat - catalytic rate constant of the enzyme.

A simple parallel reaction of SS giving rise to SL and T was written in SimBiology toolbox and solved using ode15s solver in MATLAB. The number of dntps added to SS to form SL and T is 128 and 206 respectively. Hence, the species dNTP is assumed to be a collection of 167-mers (average of 128 and 206). The equations were solved till steady state and the amount of SL and T produced were used to calculate the probability of formation of an SL or T amplicon for the entire reaction network. Since the mechanism for formation of T and PDS involves primer-based extension, the probability of formation of PDS was taken to be same as the probability of formation of T. List of parameters required to solve this kinetic model is provided in ESI Table S4. Starting

with 1.33×10^{-8} nM (100 copies) of SS, 1.19×10^{4} nM of nucleotides, 1200 nM of primer (FIP) and 124nM of enzyme (equivalent to 4 units used in LAMP reactions), the model was allowed to reach steady state, at which point 8.96×10^{-9} nM of SL and 4.34×10^{-9} nM of T was produced. The probability for formation of SL and T were hence calculated to be 0.674 and 0.326.

Supplementary Table S4. List of parameters used to calculate probability of formation of different amplicon types

S. No	Parameter name	Value	Unit	Reference
1	Volume	12.5E-6	Liter	As used in experiments
2	Starting conc of dumbbell	1.33E-8 (100 copies)	nM	As used in experiments
3	Starting conc of Primers	1200	nM	As used in experiments
4	Starting conc of Enzyme	124 (1 unit = 31 nM)	nM	Calculated
5	Starting conc of Nucleotide	2E6/167	nM	As used in the experiments
6	Primer binding rate constant (kp)	5E5	$Molarity^{-1} s^{-1}$	Mehra et al.
7	Primer unbinding rate constant (kp1)	1E-4	s^{-1}	Mehra et al.
8	Enzyme binding rate constant (ke)	1E7	$Molarity^{-1} s^{-1}$	Andreas et al
9	Enzyme Unbinding rate constant (ke1)	1E-5	s^{-1}	Andreas et al.
10	Nucleotide binding rate constant (kn)	5E5	$Molarity^{-1} s^{-1}$	Mehra et al
11	Nucleotide unbinding rate constant (kn1)	1E-4	s^{-1}	Mehra et al.
12	Catalytic rate constant of polymerase (kcat)	0.283	s^{-1}	Montagne et al

ESI Note S6 Curve fitting and extrapolation of results obtained from c-SPK model

While this model is more compact, highly parallelized and optimized, the c-SPK model also ran into issues with long processing times owing to the large number of amplicons generated in LAMP. The run times for this model were prohibitive beyond 14 minutes of reaction time for 20 starting copies of template and beyond 9 minutes of reaction time for 200 starting copies of template. While in experimental LAMP reactions all the reaction pathways occur simultaneously, in the c-SPK model only forty reactions run simultaneously. The processor has a fixed processing speed and with each iteration more data gets stored in the RAM. This leads to exponentially increasing run times with increasing reaction time (ESI Fig S16).

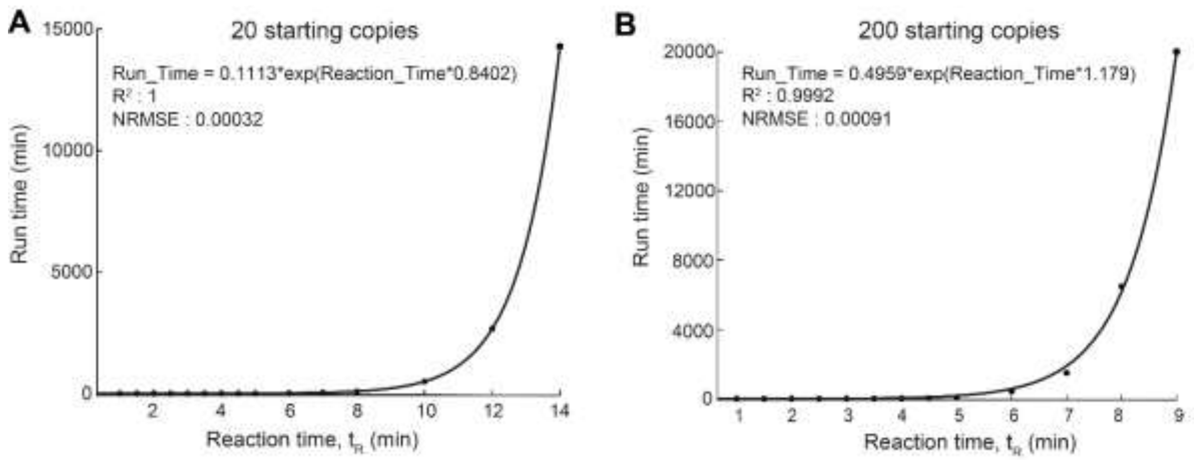

Supplementary Figure S16. Time required to run the c-SPK model for different reaction times. The run times increased exponentially with increasing reaction times. (A) 20 and (B) 200 starting copies of the dumbbell.

To overcome this hurdle and extend the model predictions to greater reaction times, a slight modification of the generalized Richard's function (Equation 1) was adopted to curve fit the amplicon concentration profiles:

$$y(t) = \frac{k}{1 + \exp((-b) * (t - m))} \qquad Equation\ 1$$

where,

y(t) represents the concentration of amplicons at time '*t*'

k represents concentration of amplicons at infinite time

b represents the maximum slope of the amplification curve, which occurs at $t = m$, *and*

m represents the time at which the growth rate is maximum

Because the reaction volume is taken to be constant for all model calculations, the model results are reported directly in terms of copies of amplicons generated. The c-SPK model was run with fixed increments of time intervals for a range of reaction times starting from 1 minute till 14 minutes and 1 minute till 9 minutes for 10 and 100 starting copies of SSD, respectively. An equal number of anti-sense single strand dumbbells (ASSD) were also considered as the template for LAMP experiments was double-stranded DNA which would lead to formation of both SSD and ASSD. Hence, the total starting number of copies of the template for model calculations was 20 and 200 copies.

The total number of amplicons generated were plotted against time for different durations of total reaction time. The formulae used for copy number calculations are explained in the previous section (ESI Note S5). Equation 1 was used for curve fitting the total amplicons plots for different reaction times. The coefficient of determination (R^2) and normalized root mean square error (NRMSE) were used to decide goodness of the fits. Fig. S17 shows representative curves for total amplicons generated in 2.5, 5 and 12 minutes of reaction time (Fig. S17A-C) with 20 starting copies of the dumbbell and 2.5, 5, and 7 minutes of reaction time (Fig. S17D-F) with 200 starting copies of the dumbbell. As was expected, the curve fitting parameters *k*, *b*, and *m* were found to

be strong functions of time. The values of *k*, *b* and *m* for different reaction times and starting concentration of dumbbell are provided in ESI Table S1 and Table S2.

Supplementary Figure S17. Representative snapshots of sigmoidal fits for total amplicons versus time as predicted by the c-SPK model.

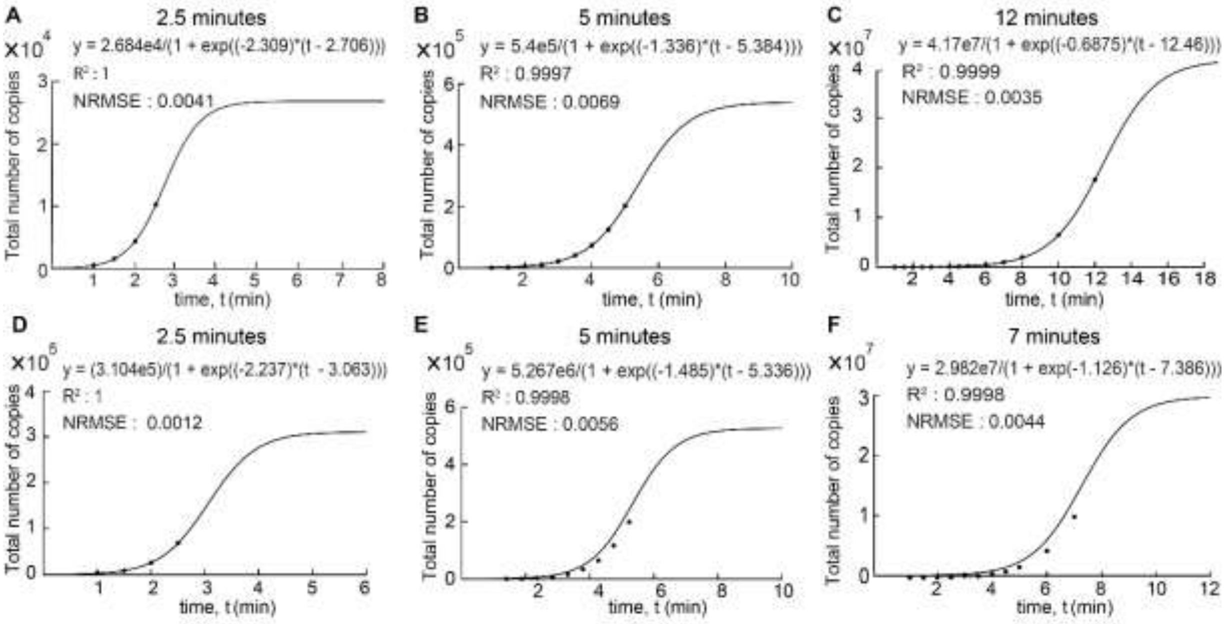

Supplementary Figure S17. Representative snapshots of sigmoidal fits for total amplicons versus time as predicted by the c-SPK model. (A), (B) and (C) for 20 starting copies of dumbbell and reaction time of 2.5, 5 and 12 minutes respectively. (D), (E) and (F) for 200 starting copies of dumbbell and reaction time of 2.5, 5 and 7 minutes respectively.

Supplementary Table S1 Variation of curve fitting parameters k, b and m for different reaction times starting with 20 copies of dumbbell.

Reaction time	k	b	m
2.5	2.68E+04	2.309	2.706
3	5.50E+04	2.048	3.218
3.5	1.20E+05	1.748	3.869
4	2.14E+05	1.566	4.415
4.5	3.19E+05	1.479	4.8
5	5.40E+05	1.336	5.384
6	1.19E+06	1.177	6.328
7	2.79E+06	1.022	7.533

8	5.67E+06	0.9139	8.654
10	1.42E+07	0.8133	10.21
12	4.17E+07	0.6875	12.46
14	1.07E8	0.595	14.73

Supplementary Table S2 Variation of curve fitting parameters k, b and m for different reaction times starting with 200 copies of dumbbell.

Reaction time	k	b	m
2.5	3.10E+05	2.237	3.063
3	5.07E+05	2.144	3.366
3.5	1.00E+06	1.944	3.859
4	1.97E+06	1.732	4.419
4.5	3.37E+06	1.592	4.902
5	5.27E+06	1.485	5.336
6	1.24E+07	1.298	6.256
7	2.98E+07	1.126	7.386
8	6.42E7	0.998	8.5

Patterns were then identified in time evolution of the curve fitting parameters present in Richard's equation (equation 1). Correlations were developed to express them as functions of time for both 20 (Fig. 18A-C) and 200 (Fig. 18D-F) starting copies of dumbbell. As defined in Richard's equation (equation 1), parameter '*k*' represents concentration of amplicons at infinite time. For c-SPK model results, since reaction volume is taken to be constant, '*k*' will represent total amplicons generated at the end of the reaction time. A power fit for '*k*' captured the trend for the model output values the best. It also generated realistic number of total amplicons at the end of reaction time when theoretically compared with number of nucleotides added in a real LAMP reaction, considering an average size for LAMP amplicons to obtain a theoretical ballpark. The LAMP reaction slows down over time due to reducing reactant concentrations and formation of longer length amplicons. Parameters '*b*' and '*m*' play a crucial role in mathematically capturing the slowing down of reaction kinetics. We chose a decreasing power fit for parameter 'b' and an

increasing power fit for parameter ‘m’ to best capture the slowing down of reaction kinetics during the later stages of the reaction.

Supplementary Figure S18. **Time-dependence of Richard’s equation parameters.**

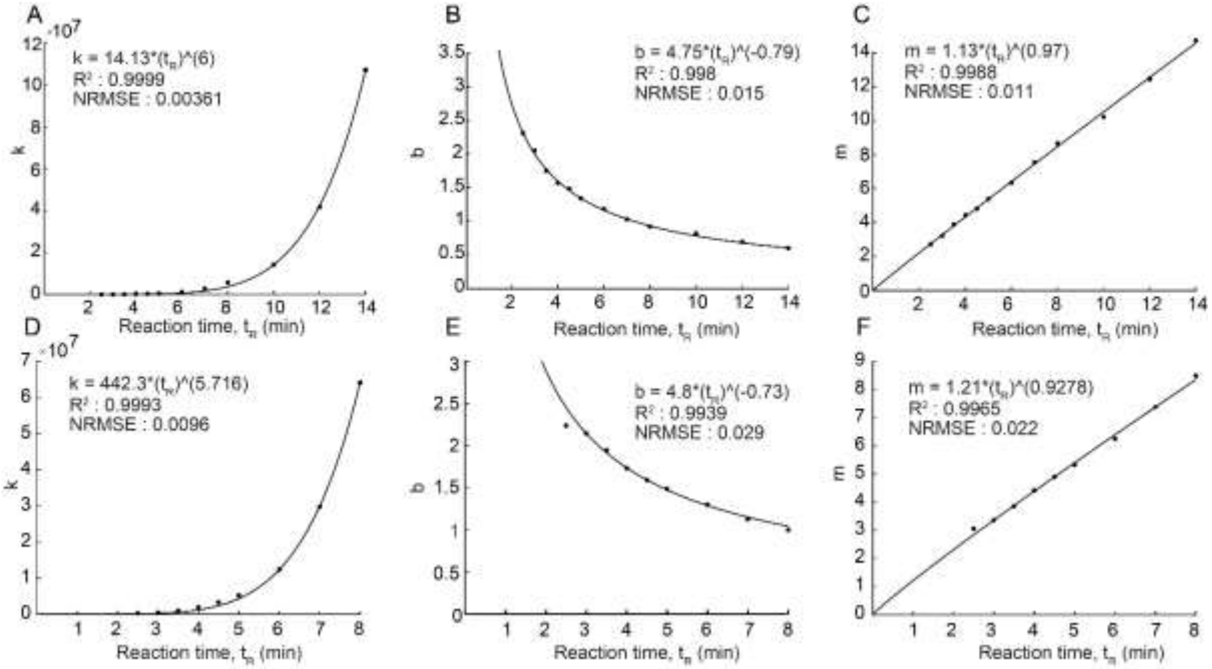

Supplementary Figure S18. **Time-dependence of Richard’s equation parameters.** A-C: Curve fitting for parameters k (A), b (B) and m (C), with 20 starting copies of the dumbbell, respectively. D-F: Curve fitting for parameters k (D), b (E) and m (F), with 200 starting copies of the dumbbell, respectively.

Extrapolating Richard’s equation parameters to generate concentration profiles for longer reaction times

As stated in assumptions (ESI NoteS2), time scale in the model comes only from nucleotide incorporation rate of enzyme (zeroth order kinetics) and the reduction of concentration of reactants does not factor into the rate due to absence of law of mass action kinetics. Accordingly, in order to incorporate the slowing down of reaction kinetics with time in the c-SPK model, the strategy was to estimate a reaction-time beyond which the maximum slope of the amplification curve would

no longer increase, i.e. estimate a cut off value for *m*. For short reaction times, *m* would increase with reaction time according to the power fit in Fig. S18 (C,F) until a critical reaction time, t_c, beyond which *m* would be kept fixed. Analogously, because *b* is the maximum slope of the amplification curve, it would also be saturated for reaction times beyond t_c. In order to determine the saturation values of *m* and *b*, the following strategy was designed:

1. *k*, *m* and *b* were calculated using their time dependent equations for 1 to 120 minutes, with an increment step of 1 minute.
2. These values were substituted in equation 1 for each minute to calculate the total number of copies (y_i) at every minute till 120 minutes.
3. In order to find the cut-off value for parameter *m*, time *t* was substituted equal to *m* in Equation 1, resulting in:

$$y(t) = \frac{\mathrm{k}}{2} \qquad\qquad - Equation\ 2$$

4. Equation 2 was used to calculate the total number of copies (y_m) at $t = m$ by substituting the value of *k* at 120 minutes, which is the saturation value at the end of 120 minutes, total reaction time considered for this illustration.
5. The value of y_m was then compared to all y_{is} to back calculate the time at which the two values were equal. The time value thus obtained is designated as the cut-off value for *m* for the 120 minutes reaction curve.
6. The *b* value corresponding to the cut-off value of *m* is defined as the cut off value of *b* for the 120 minutes reaction curve.

Total amplicon plots were generated for 20 (Fig. S19A) and 200 (Fig. S19C) starting copies of dumbbell using Equation 1 and the trends were compared with the corresponding experimental

curves with 10 (Fig. S19B) and 100 (Fig. S19D) starting copies of the double stranded genomic DNA template. For calculating the model predicted y_i values, parameter 'k' was updated at each minute up to 120 minutes while parameters 'm' and 'b' were updated till time 't'='m' minutes, beyond which their values were frozen to slow down the reaction kinetics. The rise times for the model-generated (Fig. S19A and S19C) and experimental curves (Fig. S19B and S19D) were calculated using the strategy developed by Subramanian and Gomez[1]. Briefly, the time to positive was derived as:

$$t_p = m - (2/b)$$

where,

t_p = Rise time or time to positive

m and b are parameters from Richard's equation

Supplementary Figure S19. Comparison of c-SPK model results with experimental results.

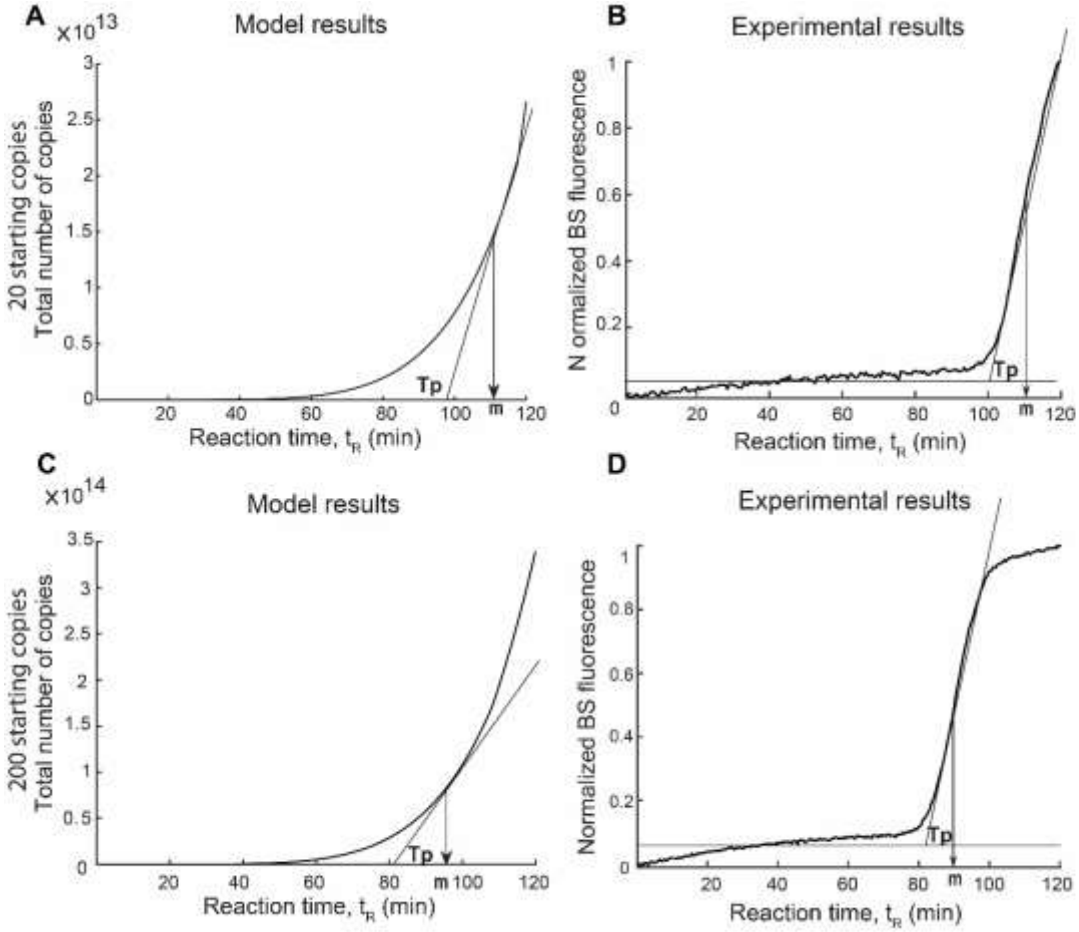

Supplementary Figure S19. Comparison of c-SPK model results with experimental results. Time dependent equations for extrapolation of k, b and m were used to generate the final amplification curves for a reaction time of 120 minutes. (A) Concentration profile of amplicons with 20 starting copies of the dumbbell (B) Real-time amplification curves for LAMP experiments with 10 starting copies of the target (double stranded *Mtb* gDNA) (C) Concentration profile of amplicons with 200 starting copies of the dumbbell. (D) Real-time amplification curves for LAMP experiments with 100 starting copies of the target (double stranded *Mtb* gDNA). BS – Background subtracted.

The model generated curves constitute of total number of amplicons plotted against reaction time while the experimental curves provide a fluorescence read-out with time, normalized between 0 to 1. Multiple strategies were tested to create a calibration curve for DNA concentration versus fluorescence in order to convert the model generated results to fluorescence read outs. But it was

found that the linear dynamic range of fluorescence read-outs was very narrow with respect to the range of DNA concentration being generated in LAMP. Furthermore, the calibration curve used by Subramanian and Gomez[1] contains only two points and considers a linear behaviour in that range. With millions of copies of amplicons being generated in LAMP, we were unsuccessful in finding a way to correlate amplicon numbers with corresponding fluorescence. Despite that, it was observed that rise time predictions were quite comparable. Rise times predicted by c-SPK model were 96.89 minutes and 81.66 minutes for 20 and 200 starting copies of the dumbbell, respectively, while the corresponding experimental rise times were 103.76 ± 7.79 minutes and 81.45 ± 2.21 minutes, respectively.

References

[1] Subramanian S, Gomez RD. An empirical approach for quantifying loop-mediated isothermal amplification (LAMP) using Escherichia coli as a model system. PLoS One 2014;9:1–10. https://doi.org/10.1371/journal.pone.0100596.
